# Transport evidence for the orbital Nernst effect

**DOI:** 10.1038/s41467-026-77878-2

**Published:** 2026-09-22

**Authors:** Wen-Yuan Li, Chun-Chi Chang, Ssu-Yen Huang, Danru Qu

**Affiliations:** 1 Department of Physics, National Taiwan University, Taipei, Taiwan; 2 Center for Condensed Matter Sciences, National Taiwan University, Taipei, Taiwan; 3 Center of Atomic Initiatives for New Materials, National Taiwan University, Taipei, Taiwan

**Keywords:** Spintronics, Surfaces, interfaces and thin films

## Abstract

Orbital Hall effects have recently attracted significant attention for generating substantial orbital angular momentum currents in both strong- and weak-spin-orbit-coupling materials. Despite extensive theoretical and experimental progress on the orbital Hall effect, its thermal counterpart, in which a temperature gradient drives a transverse orbital current, known as the orbital Nernst effect, has remained experimentally elusive. Here, we report the first experimental evidence for the orbital Nernst effect by measuring the magneto-thermopower in the yttrium iron garnet (YIG)/Pt/CuO_x_ heterostructures. Through systematic comparison with the YIG/Pt and YIG/CuO_x_ heterostructures, we show that both the magneto-thermopower and the magnetoresistance for YIG/Pt/CuO_x_ are markedly enhanced due to the contributions from the orbital Nernst effect and the orbital Hall effect in CuO_x_, respectively. Our experimental evidence for the orbital Nernst effect supports the orbital degree of freedom as a promising route for heat-energy harvesting and orbital angular momentum thermal transport in earth-abundant materials.

## Abstract (web summary)

The authors report experimental evidence of the orbital Nernst effect, demonstrating that heat can generate transverse orbital currents in a copper oxide film and opening new opportunities for heat-driven orbitronics.

## Introduction

Orbitronics, which exploits orbital angular momentum, has recently emerged as a promising platform for next-generation information technologies. The generation of orbital currents does not rely on spin-orbit coupling (SOC) and is typically more efficient than that of spin currents^1–3^. It broadens the range of viable materials, provides greater flexibility, and offers a powerful and effective alternative for manipulating angular momentum in condensed-matter systems.

The key mechanisms that generate orbital angular momentum are the orbital Hall effect (OHE) and the orbital Rashba-Edelstein effect (ORE), in which a charge current generates a transverse orbital angular momentum flow through a momentum-space orbital texture^4^. However, despite substantial efforts to detect the OHE, its thermal counterpart, the orbital Nernst effect (ONE), or the orbital Rashba-Edelstein Nernst effect (ORNE), in which a transverse orbital current arises from a heat current^3^, has not yet been experimentally established. The demonstration of the ONE would therefore mark a critical milestone in orbitronics, establishing heat-driven orbital transport as a new fundamental physical phenomenon, and providing a new framework for thermoelectric energy conversion through orbital degree of freedom.

**Fig. 1:**
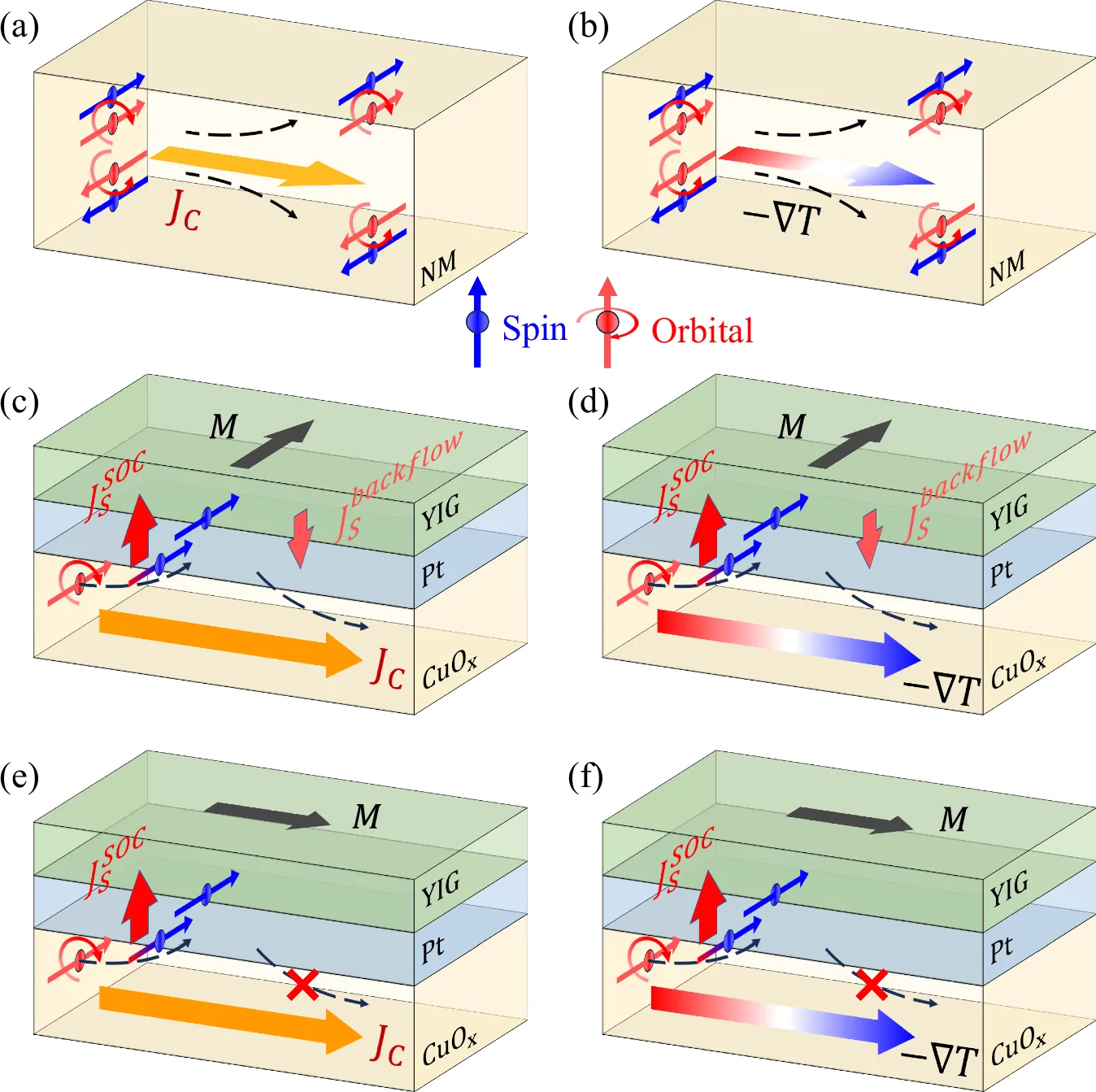
Schematic illustration of the electrical and thermal spin and orbital current generations. **a** Spin Hall and orbital Hall effects upon a charge current injection (orange arrow) in a nonmagnetic metal (NM), producing transverse spin (blue arrows) and orbital (red circular arrows) currents. **b** Spin Nernst and orbital Nernst effects upon a temperature gradient (red-to-blue arrow) in NM. **c**–**f** Schematic of the magnetoresistance (**c**, **e**) and magneto-thermopower (**d**, **f**) measurements in the YIG/Pt/CuO_x_ heterostructure. The orbital current generated in CuO_x_ is converted into an additional interfacial spin current ${J}_{S}^{{SOC}}$ (red arrow) via the spin-orbit coupling of Pt. Depending on the relative orientation between the spin ${{\boldsymbol{\sigma }}}$ and the YIG magnetization **M** (black arrow), the total spin current at the YIG/Pt interface is either reflected (**c**, **d**) when ${{\boldsymbol{\sigma }}}\parallel {{\bf{M}}}$, generating a backflow spin current ${J}_{S}^{{backflow}}$ that is converted into a detectable voltage signal via the inverse spin Hall effect, or absorbed (**e**, **f**) when ${{\boldsymbol{\sigma }}}\perp {{\bf{M}}}$, in which case no backflow spin current is generated, as indicated by the red cross.

As illustrated in Fig. 1a, in the case of OHE, a charge current in a nonmagnetic material generates a transverse orbital angular momentum flow (red arrow) through a momentum-space orbital texture^4^. In ORE, the momentum-space orbital texture is induced at the interface and is often observed between an oxide layer and a metallic layer^5,6^. Notably, the emergence of orbital texture is not restricted to idealized interface geometries. As discussed in ref. ^7^, in naturally oxidized Cu, where oxygen is distributed non-uniformly, the key ingredient for generating orbital texture is the local Cu-O hybridization rather than a well-defined interface. Although the detailed physical mechanisms for OHE and ORE differ, they both, phenomenologically, induce a transverse orbital angular momentum flow upon the injection of a charge current. For simplicity, ORE is implicitly included in the following discussion of OHE.

The OHE is analogous to the spin Hall effect (SHE), where a charge current generates a transverse spin current flow (blue arrow in Fig. 1a). In contrast to the SHE, the OHE does not require any SOC. Since both the OHE and SHE share the same detection geometries, distinguishing between them experimentally can be challenging. However, this difficulty can be mitigated by choosing appropriate materials, such as light metals CuO_x_^5,6^ and Ti^8^, which have negligible SOC, or materials with opposite signs for OHE and SHE, such as Ta^9^. Experimentally, the OHE has been detected directly via optical Kerr rotation^10^ and indirectly via SOC-mediated conversion into a spin current, manifesting as an additional torque^6^, a modified magnetoresistance^5^, or a nonlocal voltage^11^.

In materials with orbital texture in the bulk or at the interface, a transverse orbital current is also expected to arise through the ONE or ORNE upon a heat current flow^3^, as shown in Fig. 1b (red arrow). For simplicity, in the following discussion, the orbital angular momentum generated from the ORNE is also implicitly included in the discussion of ONE. Accompanying the ONE is the spin Nernst effect (SNE), which generates a transverse spin current upon a heat current (Fig. 1b, blue arrow). While the SNE has been experimentally observed in heavy metals^12–15^, topological insulators^16^, and alloys^17^, the ONE, which is predicted to be more significant than SNE^3^, has been missing. Demonstrating the ONE would therefore mark a critical milestone in orbitronics.

Here, we report the transport evidence for the ONE in the light metal CuO_x_. Previous studies have revealed strong *p*-*d* hybridization at the Cu-O interface, which gives rise to an orbital angular momentum texture and a large orbital Hall conductivity in CuO_x_^7^. Meanwhile, owing to the weak SOC in CuO_x_, the generation of spin current is minimal, allowing for the efficient detection of the orbital current. We thus incorporate CuO_x_ into the ferromagnetic insulator yttrium iron garnet (YIG)/Pt/CuO_x_ heterostructure, which is further protected by a SiO_2_ layer, and study its electrical and thermal transport properties. The use of an insulating ferromagnet eliminates any anisotropic magnetoresistance (AMR) or anomalous Nernst (ANE) contributions, ensuring that the observed signal arises solely from the nonmagnetic metallic layers.

For the YIG/Pt/CuO_x_ heterostructure, upon a charge current flow (${J}_{C}$), as shown in Fig. 1c, e, or under a temperature gradient (∇T), as shown in Fig. 1d, f, a transverse spin current is generated in the Pt layer through the SHE or SNE, respectively. In addition, a transverse orbital current is expected to be generated from the CuO_x_ layer via the OHE and ONE. The orbital current induced in CuO_x_ then injects into the Pt layer, is converted into additional spin current through the SOC of Pt, and modifies the spin accumulation at the YIG/Pt interface. Depending on the relative orientations between spin (σ) and YIG magnetization (M), the total spin current is either reflected (Fig. 1c, d, $\sigma {||}M$) or absorbed (Fig. 1e, f, $\sigma \perp M$). The reflected spin current further converts back to a charge current through the inverse spin Hall effect (ISHE) in the Pt layer. The spin and orbital currents can thus be detected as changes in the magnetoresistance^18^ and magneto-thermopower^17^. By comparing the heterostructures with and without a CuO_x_ layer, we could conclusively isolate the orbital contribution originating from CuO_x_.

## Results and discussion

We prepare three types of samples, the YIG/Pt(3)/CuO_x_(2), YIG/Pt(3), and YIG/CuO_x_(5) samples, each capped with a 2-nm-thick SiO_2_ layer, to investigate and isolate the orbital current contributions. The numbers in parentheses denote the nominal thicknesses in nanometers. The polycrystalline YIG slabs are used as substrates; they have a typical width of 2 mm, a length of 10 mm, and a thickness of 0.5 mm. The transition metal layers are deposited onto the YIG substrate by DC magnetron sputtering in a vacuum chamber with a base pressure better than $1\times {10}^{-7}$ Torr. After deposition of the Cu layer, the sample is removed from the chamber and naturally oxidized in air for 2 days. Based on a prior report^19^, the naturally oxidized CuO_x_ layer does not form distinct Cu/Cu_2_O/CuO interfaces, but exhibits a gradual depth-dependent oxygen distribution. After oxidation, a SiO_2_ capping layer is deposited onto CuO_x_ through RF sputtering to prevent further oxidation.

We first present magneto-thermopower measurements to reveal its thermally induced orbital current via the ONE. Details of the experimental conditions are discussed in Supplementary Note 1. Unlike the charge current that flows only within the conducting film plane, the heat current can flow both in-plane and out-of-plane within the whole sample. For YIG-based samples, the in-plane heat current generates an MR-like signal that is symmetric with respect to the magnetic field, known as the magneto-thermopower. In contrast, the out-of-plane heat current generates an anomalous Hall-like signal that is antisymmetric with respect to the magnetic field, due to the spin Seebeck effect of YIG. Through symmetry analysis, we can isolate signals induced solely by the in-plane heat current [see Supplementary Note 2].

**Fig. 2:**
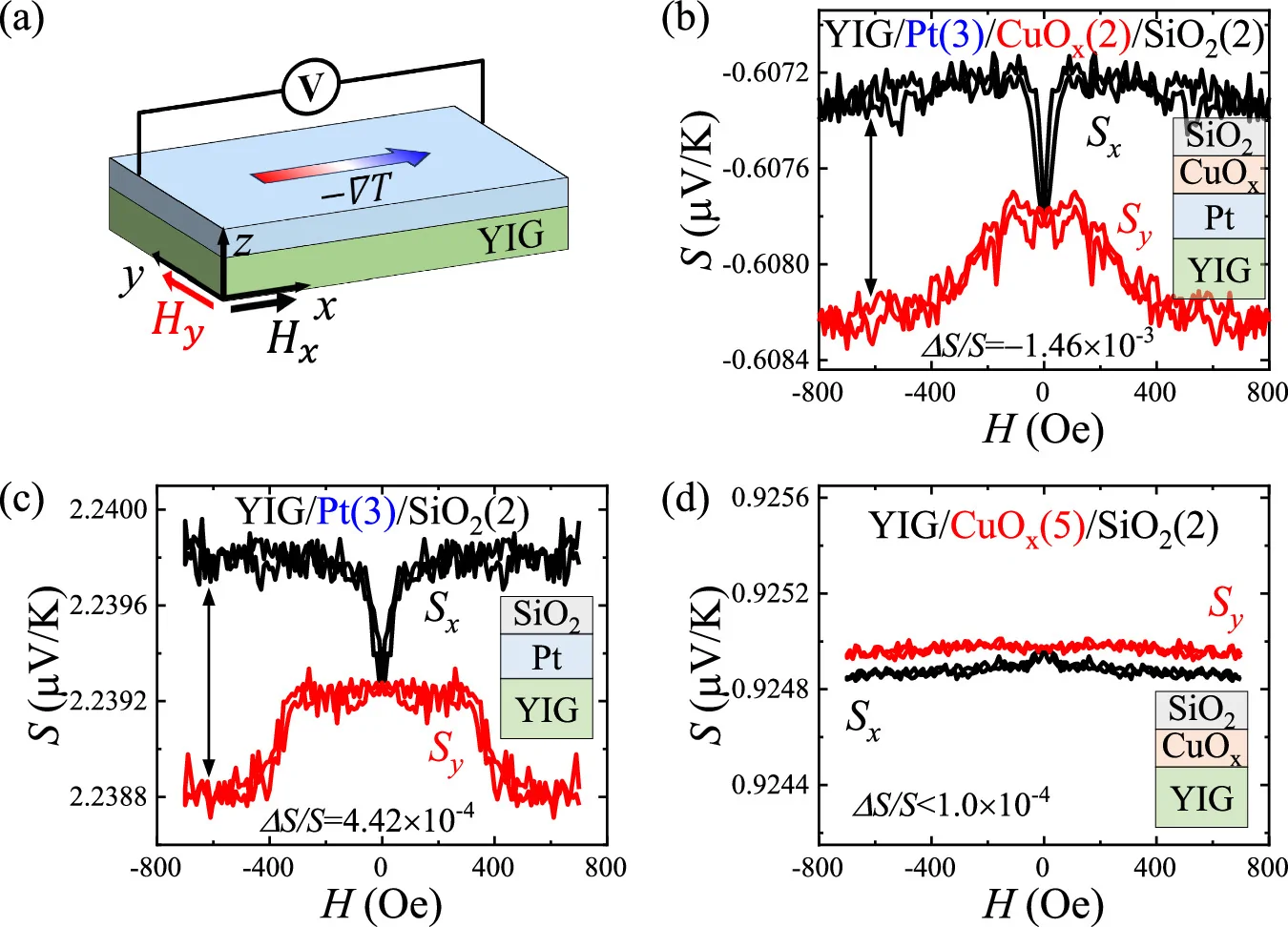
The spin and orbital Nernst magneto-thermopower measurements. **a** Schematic illustration of the measurement geometry: a temperature gradient −∇T is applied along the *x*-axis, the longitudinal voltage V is measured along the same direction, and the in-plane magnetic field H is swept along the *x*-axis (${H}_{x}$) or *y*-axis (${H}_{y}$). **b**–**d** Magnetic field-dependent Seebeck coefficient *S* for **b** YIG/Pt(3)/CuO_x_(2)/SiO_2_(2), **c** YIG/Pt(3)/SiO_2_(2), and **d** YIG/CuO_x_(5)/SiO_2_(2). Black and red curves represent the Seebeck coefficient obtained with the magnetic field applied along the *x*-axis (${S}_{x}$) and *y*-axis (${S}_{y}$), respectively. The magneto-thermopower ratio ΔS/S is defined as $({S}_{x}-{S}_{y})/{S}_{x}$.

As shown in Fig. 2a, a heat flow with $\nabla T=1.9\pm 0.1\ {{{\rm{mm}}}/{{\rm{K}}}}$ is applied along the *x*-axis, and the Seebeck voltage, denoted as ${V}_{x}$ and ${V}_{y}$, is measured under an external magnetic field (H) applied along the *x*- (${H}_{x}$) and *y*- (${H}_{y}$) axes, respectively. The magnetic field sweeps from 700 Oe to −700 Oe and reverses. This field range is sufficient to align the YIG magnetization M along the in-plane field direction [see Supplementary Note 3]. The Seebeck coefficient S=−V/ΔT obtained under ${H}_{x}$ and ${H}_{y}$ are denoted as ${S}_{x}$ and ${S}_{y}$, respectively. To obtain the Seebeck coefficient for the thin film, contributions from the connecting Cu wires (${S}_{{\rm{Cu}}}=+ 1.68\ {{\rm{\mu }}}{{\rm{V}}}/{{\rm{K}}}$), must be subtracted^17^ [see Supplementary Note 4]. In the following discussion, the Seebeck voltage obtained has already been corrected, excluding the contributions from the bonding wires. We also average the thermopower signals over 50 measurement cycles to improve the signal-to-noise ratio. All measurements are conducted at room temperature.

Figure 2b–d shows the spin and orbital Nernst magneto-thermopower (SNMT and ONMT) results for the YIG/Pt(3), YIG/Pt(3)/CuO_x_(2), and YIG/CuO_x_(5) samples originating from the in-plane heat current. Within the same experimental resolution, only the YIG/Pt and YIG/Pt/CuO_x_, but not the YIG/CuO_x_ samples, show sizable differences between ${S}_{x}$ and ${S}_{y}$. Notably, the difference in the saturation field for ${S}_{x}$ and ${S}_{y}$ originates from the anisotropy in the YIG magnetization under ${H}_{x}$ and ${H}_{y}$ [see Supplementary Note 3]. Since no ferromagnetic metal layer is used in our study, the difference between ${S}_{x}$ and ${S}_{y}$ is attributed to the spin current absorption and reflection at the YIG/Pt interface. The sizable and negligible values of $\varDelta S={S}_{x}-{S}_{y}$ for YIG/Pt and YIG/CuO_x_, respectively, indicate a sizable spin Nernst effect in Pt but not in Cu nor CuO_x_.

For YIG/Pt(3), which includes the contribution from the SNE, we obtain the SNMT signal through the magneto-thermopower measurement, as shown in Fig. 2c. We extract ${S}_{{{\rm{Pt}}}}=2.240\pm 0.006\ {{\rm{\mu }}}{{\rm{V}}}/{{\rm{K}}}$,  $\varDelta S=0.99\pm 0.07\ {{\rm{nV}}}/{{\rm{K}}}$, and a SNMT ratio $\varDelta S/S=(4.42\pm 0.31)\times {10}^{-4}$ at $H=-700\ {{\rm{Oe}}}$. Using the SNMT model^12^, the magneto-thermopower ratio is expressed as

1 $$\frac{\Delta S}{S}=-{\theta }_{{{\rm{SN}}}}{\theta }_{{{\rm{SH}}}}\frac{{\lambda }_{S}}{{t}_{{{\rm{Pt}}}}}\frac{2{\lambda }_{S}{\rho }_{{{\rm{Pt}}}}{G}_{{{\rm{r}}}}{\tanh }^{2}\frac{{t}_{{{\rm{Pt}}}}}{2{\lambda }_{S}}}{1+2{\lambda }_{S}{\rho }_{{{\rm{Pt}}}}{G}_{{{\rm{r}}}}\coth \frac{{t}_{{{\rm{Pt}}}}}{{\lambda }_{S}}}$$

By using the Pt spin diffusion length ${\lambda }_{S}=1.5\ {{\rm{nm}}}$ ^12^, spin-mixing conductance ${G}_{{\rm{r}}}=4\times {10}^{14}\ {\Omega }^{-1}{{{\rm{m}}}}^{-2}$ ^12^ obtained from literature, and Seebeck coefficient ${S}_{{{\rm{Pt}}}}=2.24\ {{\rm{\mu }}}{{\rm{V}}}/{{\rm{K}}}$, magneto-thermopower ratio $\frac{\Delta {S}_{{{\rm{Pt}}}}}{{S}_{{{\rm{Pt}}}}}=4.42\times {10}^{-4}$, resistivity ${\rho }_{{{\rm{Pt}}}}=60.8\ {{\rm{\mu }}}\Omega \cdot {{\rm{cm}}}$, spin Hall angle ${\theta }_{{{\rm{SH}}},{{\rm{Pt}}}}=2.9\pm 0.1\%$, obtained from this study (some of these parameters are discussed later), we extract a spin Nernst angle ${\theta }_{{{\rm{SN}}}}=-12.5\pm 1.0\%$ for Pt. The corresponding spin Nernst conductivity is found to be ${\alpha }_{{{\rm{SN}}}}=-0.23\pm 0.02\ (\hslash /e)({{\rm{A}}}/{{\rm{m}}}\cdot {{\rm{K}}})$, which is consistent with our previous report^17^.

For YIG/Pt(3)/CuO_x_(2), as shown in Fig. 2b, we find that the Seebeck coefficient for the whole sample ${S}_{{tot}}$ changes sign and becomes ${S}_{{tot}}=-0.608\pm 0.009\ {{\rm{\mu }}}{{\rm{V}}}/{{\rm{K}}}$. The sign change is caused by the opposite Seebeck coefficient for thin films of Pt and CuO_x_. Using a parallel-resistance model, $\frac{{S}_{{tot}}}{{R}_{{tot}}}=\frac{{S}_{{{\rm{Pt}}}}}{{R}_{{{\rm{Pt}}}}}+\frac{{S}_{{{\rm{Cu}}}{{{\rm{O}}}}_{{{\rm{x}}}}}}{{R}_{{{\rm{Cu}}}{{{\rm{O}}}}_{{{\rm{x}}}}}}$, with the experimentally obtained sheet resistance ${R}_{{tot}}=111.6\ \Omega$ for the whole sample, ${R}_{{{\rm{Pt}}}}=202.5\ \Omega$ for the Pt layer, and ${R}_{{{\rm{Cu}}}{{{\rm{O}}}}_{{{\rm{x}}}}}=248.5\ \Omega$ (discussed later) for the CuO_x_ layer, we estimate the Seebeck coefficient for the 2-nm-thick CuO_x_ as ${S}_{{{\rm{Cu}}}{{{\rm{O}}}}_{{{\rm{x}}}}}=-4.10\ {{\rm{\mu }}}{{\rm{V}}}/{{\rm{K}}}$, which shows the opposite sign to ${S}_{{{\rm{Pt}}}}$. We also note that the sign of ${S}_{{{\rm{Cu}}}{{{\rm{O}}}}_{{{\rm{x}}}}}$ is opposite for the 2-nm-thick and 5-nm-thick CuO_x_ layers in Fig. 2b, d. Such sign reversals have been previously reported in thin-film systems^20^ and are attributed to enhanced surface scattering when the film thickness approaches the electron mean free path^21^. For the YIG/Pt/CuO_x_ sample, the Seebeck coefficient difference is $\varDelta S=0.89\pm 0.09\ {{\rm{nV}}}/{{\rm{K}}}$, and the ratio is $\varDelta S/S=(-1.46\pm 0.09)\times {10}^{-3}$. If one assumes CuO_x_ layer contributes only a shunting effect, the effective ΔS/S for Pt can be estimated as $\frac{\Delta {S}_{{{\rm{Pt}}}}}{{S}_{{{\rm{Pt}}}}} \sim \frac{{R}_{{{\rm{Pt}}}}[{R}_{{tot}}\Delta {S}_{{tot}}+\left({S}_{{{\rm{Pt}}}}-{S}_{{tot}}\right)\Delta {R}_{{tot}}]}{{R}_{{tot}}^{2}{S}_{{{\rm{Pt}}}}} \sim (1.09\pm 0.12)\times {10}^{-3}$ [see Supplementary Note 5]. This value is about twice that obtained for YIG/Pt. Therefore, the strongly enhanced spin-charge interconversion cannot be attributed solely to shunting and must originate from the ONE generated in the CuO_x_ layer.

To quantitatively analyze the orbital current generated by the CuO_x_ layer, we apply a simplified drift-diffusion model. In this framework, the orbital current produced in CuO_x_ is converted into a spin current in Pt through SOC and subsequently diffuses toward the YIG/Pt interface. Depending on the magnetization orientation of YIG, the spin current is either reflected (for $M\parallel \sigma$) or absorbed (for $M\perp \sigma$), and the reflected spin current is detected as a voltage change through the ISHE in Pt. In the model, we make the following assumptions (i) immediate orbital-to-spin conversion at the Pt/CuO_x_ interface, (ii) negligible inverse orbital Hall effect (IOHE) in CuO_x_, and (iii) strong spin relaxation within Pt due to its short spin diffusion length (${\lambda }_{S}=1.5\ {{\rm{nm}}}$). Combining the spin current generated by Pt and taking these assumptions into account, we have

2 $$\frac{{S}_{{{\rm{Pt}}}}(m\parallel x)-{S}_{{{\rm{Pt}}}}(m\parallel y)}{{S}_{{{\rm{Pt}}}}}=-	 \left({\theta}_{{{\rm{SN}}},{{\rm{Pt}}}}\tanh \frac{{t}_{{{\rm{Pt}}}}}{2{\lambda }_{S}}+\frac{{\rho }_{{{\rm{Pt}}}}{S}_{{{\rm{Cu}}}{{{\rm{O}}}}_{{{\rm{x}}}}}}{{\rho }_{{{\rm{Cu}}}{{{\rm{O}}}}_{{{\rm{x}}}}}{S}_{{{\rm{Pt}}}}}{\theta }_{O{{\rm{N}}},{{{\rm{CuO}}}}_{{{\rm{x}}}}}{{\rm{csch}}}\frac{{t}_{{{\rm{Pt}}}}}{{\lambda }_{S}}\right)\\ 	 {\theta }_{{{\rm{SH}}},{{\rm{Pt}}}}\frac{{\lambda }_{S}}{{t}_{{{\rm{Pt}}}}}\frac{2{\lambda }_{S}{\rho }_{{{\rm{Pt}}}}{G}_{{{\rm{r}}}}\tanh \frac{{t}_{{{\rm{Pt}}}}}{2{\lambda }_{S}}}{1+2{\lambda }_{S}{\rho }_{{{\rm{Pt}}}}{G}_{{{\rm{r}}}}\coth \frac{{t}_{{{\rm{Pt}}}}}{{\lambda }_{S}}}$$

which we used to estimate the orbital Nernst angle of CuO_x_ [see Supplementary Note 6]. The expression in parentheses represents the total heat-induced spin current reaching the YIG/Pt interface, where the first term corresponds to the spin Nernst current generated by Pt and the second term corresponds to the SOC-mediated orbital Nernst current generated by CuO_x_.

Using the Pt spin diffusion length ${\lambda }_{S}=1.5\ {{\rm{nm}}}$ ^12^, spin-mixing conductance ${G}_{{\rm{r}}}=4\times {10}^{14}\ {\Omega }^{-1}{{{\rm{m}}}}^{-2}$ ^12^ obtained from literature, ${R}_{{{\rm{Pt}}}}=202.5\ \Omega$, ${R}_{{{\rm{Cu}}}{{{\rm{O}}}}_{{{\rm{x}}}}}=248.5\ \Omega$, ${S}_{{{\rm{Pt}}}}=2.24\ {{\rm{\mu }}}{{\rm{V}}}/{{\rm{K}}}$, ${S}_{{{\rm{Cu}}}{{{\rm{O}}}}_{{{\rm{x}}}}}=-4.10\ {{\rm{\mu }}}{{\rm{V}}}/{{\rm{K}}}$, $\frac{\Delta {S}_{{{\rm{Pt}}}}}{{S}_{{{\rm{Pt}}}}}=1.09\times {10}^{-3}$, ${\rho }_{{{\rm{Pt}}}}=60.8\ {{\rm{\mu }}}\Omega \cdot {{\rm{cm}}}$, ${\theta }_{{{\rm{SH}}},{{\rm{Pt}}}}=2.9\%$, and ${\theta }_{{{\rm{SN}}},{{\rm{Pt}}}}=-12.5\%$ obtained from this study (some of these parameters are discussed later) we estimate ${\theta }_{{{\rm{ON}}},{{\rm{Cu}}}{{{\rm{O}}}}_{{{\rm{x}}}}}=+ 22.5\pm 6.8\%$, with the orbital Nernst conductivity for CuO_x_ estimated as ${\alpha }_{{{\rm{ON}}},{{{\rm{CuO}}}}_{{{\rm{x}}}}}=-0.93\pm 0.34\ (\hslash /e)({{\rm{A}}}/{{\rm{m}}}\cdot {{\rm{K}}})$. The substantial enhancement of the effective magneto-thermopower ratio in the YIG/Pt/CuO_x_ sample, therefore, provides strong evidence for the existence of the ONE in CuO_x_.

It is worth noting that we do not distinguish between the orbital Nernst effect originating from Cu and the orbital Rashba–Edelstein effect induced by the Cu-O hybridization. For simplicity, we treat the naturally oxidized CuO_x_ as an effective single layer that produces the orbital current via both mechanisms. A controlled study on the oxidation of the Cu layer would be useful for further separating the two effects.

**Fig. 3:**
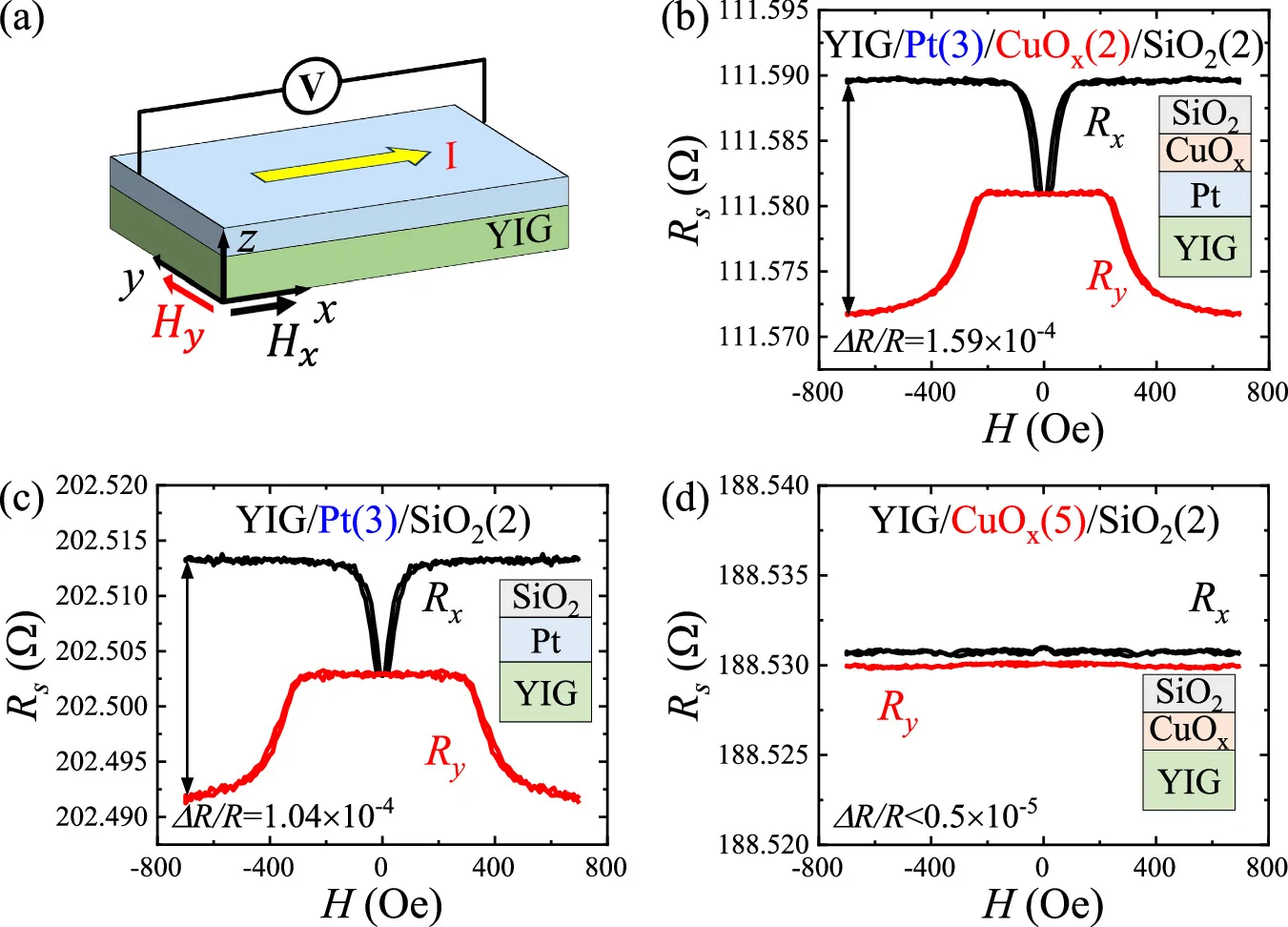
The spin Hall and orbital Hall magnetoresistance measurements. **a** Schematic illustration of the measurement geometry: a charge current I is applied along the *x*-axis, the longitudinal voltage V is measured along the same direction, and the in-plane magnetic field H is swept along the *x*-axis (${H}_{x}$) or *y*-axis (${H}_{y}$). **b**–**d** Magnetic field-dependent sheet resistance ${R}_{{{\rm{s}}}}$ for **b** YIG/Pt(3)/CuO_x_(2)/SiO_2_(2), **c** YIG/Pt(3)/SiO_2_(2), and **d** YIG/CuO_x_(5)/SiO_2_(2). Black and red curves represent the sheet resistance measured with the magnetic field applied along the *x*-axis (${R}_{x}$) and *y*-axis (${R}_{y}$), respectively. The magnetoresistance ratio ΔR/R is defined as $({R}_{x}-{R}_{y})/{R}_{x}$.

After confirming the thermally induced orbital current in CuO_x_, we perform spin Hall magnetoresistance (SMR) measurements on the same samples to verify and probe the electrically induced orbital current via the OHE. The resistance (R) is obtained using a two-probe method, where the contact resistance is negligible compared to the thin-film resistance. We use the normalized sheet resistance, defined as ${R}_{s}={Rw}/l$, in the following discussion, to provide a direct comparison among different samples. As shown in Fig. 3a, in the magnetoresistance (MR) measurement, a charge current of 10 mA is applied along the *x*-axis, and a voltage is measured along the same direction. The magnetic field sweeps from 700 Oe to −700 Oe and reverses. The sheet resistance ${R}_{x}$ and ${R}_{y}$ are the longitudinal MR obtained under ${H}_{x}$ and ${H}_{y}$, respectively. To improve the signal-to-noise ratio, the resistance is averaged over 20 measurement cycles.

Figure 3b–d shows the field-dependent MR for the three samples. For both YIG/Pt/CuO_x_ and YIG/Pt, shown in Fig. 3b, c, they exhibit the SMR features with ${R}_{x} > {R}_{y}$. Since no ferromagnetic metal layer is used in our study, the sizable difference between ${R}_{x}$ and ${R}_{y}$ originates from the spin current reflection or absorption at or near the YIG/Pt interface.

For the case of YIG/Pt(3), as shown in Fig. 3c, the spin current originates from the SHE in Pt. The sheet resistance of the sample is ${R}_{S}=202.5\pm 9.1\ \Omega$, the resistance difference is $\Delta {R}_{S}={R}_{x}-{R}_{y}=0.021\pm 0.001\ \Omega$ and the MR ratio is $\Delta {R}_{S}/{R}_{S}=\left(1.04\pm 0.05\right)\times {10}^{-4}$ under the saturation magnetic field of $H=-700\ {{\rm{Oe}}}$. By considering the Pt spin diffusion length ${\lambda }_{S}=1.5\ {{\rm{nm}}}$ ^12^, spin-mixing conductance ${G}_{{\rm{r}}}=4\times {10}^{14}\ {\Omega }^{-1}{{{\rm{m}}}}^{-2}$ ^12^ obtained from literature, and using the resistivity ${\rho }_{{{\rm{Pt}}}}=60.8\pm 2.7\ {{\rm{\mu }}}\Omega \cdot {{\rm{cm}}}$, thickness ${t}_{{\rm{Pt}}}=3$ nm obtained from this study, by fitting with the SMR model^22^

3 $$\frac{\Delta R}{R}={\theta }_{{\rm{SH}}}^{2}\frac{{\lambda }_{S}}{{t}_{{\rm{Pt}}}}\frac{2{\lambda }_{S}{\rho }_{{\rm{Pt}}}{G}_{{\rm{r}}}{\tanh }^{2}\frac{{t}_{{\rm{Pt}}}}{2{\lambda }_{S}}}{1+2{\lambda }_{S}{\rho }_{{\rm{Pt}}}{G}_{\rm{{r}}}\coth \frac{{t}_{{\rm{Pt}}}}{{\lambda }_{S}}}$$

we obtain the spin Hall angle of Pt as ${\theta }_{{\rm{SH}}}=+ 2.9\pm 0.1\%$, which is consistent with previous studies^17^.

For the case of YIG/Pt(3)/CuO_x_(2), the sheet resistance is reduced to ${R}_{S}=111.6\pm 6.7\ \Omega$ due to the conducting CuO_x_, the resistance difference is $\Delta {R}_{S}=0.018\pm 0.001\ \Omega$, and the MR ratio is $\Delta {R}_{S}/{R}_{S}=(1.59\pm 0.10)\times {10}^{-4}$. Using a parallel-resistance model, $\frac{1}{{R}_{\parallel }}=\frac{1}{{R}_{{{\rm{Pt}}}}}+\frac{1}{{R}_{{{\rm{Cu}}}{{{\rm{O}}}}_{{{\rm{x}}}}}}$, and with the ${R}_{{{\rm{Pt}}}}=202.5\ \Omega$, we estimate the resistance for the 2-nm-thick CuO_x_ as ${R}_{{{\rm{Cu}}}{{{\rm{O}}}}_{{{\rm{x}}}}}=248.5\pm 35.7\ \Omega$. Assuming CuO_x_ contributes only a shunting effect, the effective MR ratio for the Pt layer is estimated as $\frac{\Delta {R}_{{{\rm{Pt}}}}}{{R}_{{{\rm{Pt}}}}}\sim \frac{\Delta {R}_{\parallel }{R}_{{{\rm{Pt}}}}}{{R}_{\parallel }^{2}} \sim (2.88\pm 0.45)\times {10}^{-4}$. The MR ratio is enhanced by nearly a factor of three relative to the reference Pt sample, demonstrating that the signal cannot be explained solely by SHE in Pt, consistent with the behavior in the magneto-thermopower measurements. This pronounced enhancement clearly indicates that an additional angular-momentum contribution from the CuO_x_ layer must be considered.

We exclude the spin Hall contribution from CuO_x_ using the YIG/CuO_x_(5) sample. As shown in Fig. 3d, within the same experimental resolution and with a comparable sheet resistance of ${R}_{S}$ = 188.5 Ω, a negligible MR ratio with $\Delta {R}_{S}/{R}_{S} < 5\times {10}^{-6}$ is observed for YIG/CuO_x_, indicating that CuO_x_ itself cannot efficiently generate a spin current to account for the enhanced MR for the YIG/Pt/CuO_x_ sample. We also exclude the contribution from the Pt/Cu interface [see Supplementary Note 7]. The enhanced signal is therefore attributed to the generation of orbital angular momentum in the CuO_x_ film, which is further converted into a spin current through the SOC in Pt.

Using similar assumptions and the drift-diffusion model mentioned earlier, the effective MR ratio of Pt can be expressed as [see Supplementary Note 6]

4 $$\frac{{R}_{{{\rm{Pt}}}}\left({m||x}\right)-{R}_{{{\rm{Pt}}}}\left({m||y}\right)}{{R}_{{{\rm{Pt}}}}}=	 \left({\theta }_{{{\rm{SH}}},{{\rm{Pt}}}}\tanh \frac{{t}_{{{\rm{Pt}}}}}{2{\lambda }_{S}}+\frac{{\rho }_{{{\rm{Pt}}}}}{{\rho }_{{{\rm{Cu}}}{{{\rm{O}}}}_{{{\rm{x}}}}}}{\theta }_{{{\rm{OH}}},{{\rm{Cu}}}{{\rm{O}}}_{{\rm{x}}}}{{\rm{csch}}}\frac{{t}_{{{\rm{Pt}}}}}{{\lambda }_{S}}\right)\\ 	 {\theta }_{{{\rm{SH}}},{{\rm{Pt}}}}\frac{{\lambda }_{S}}{{t}_{{{\rm{Pt}}}}}\frac{2{\lambda }_{S}{\rho }_{{{\rm{Pt}}}}{G}_{{{\rm{r}}}}\tanh \frac{{t}_{{{\rm{Pt}}}}}{2{\lambda }_{S}}}{1+2{\lambda }_{S}{\rho }_{{{\rm{Pt}}}}{G}_{{{\rm{r}}}}\coth \frac{{t}_{{{\rm{Pt}}}}}{{\lambda }_{S}}}$$

The expressions in parentheses represent the total spin current reaching the YIG/Pt interface, where the first term is the spin current generated by the SHE in Pt and the second term is the SOC-mediated orbital current generated by the OHE in the CuO_x_. By substituting ${\lambda }_{S}=1.5\ {{\rm{nm}}}$, ${G}_{{\rm{r}}}=4\times {10}^{14}\ {\Omega }^{-1}{{{\rm{m}}}}^{-2}$ obtained from literature^12^, and the ${R}_{{{\rm{Pt}}}}=202.5\ \Omega$, ${R}_{{{\rm{Cu}}}{{{\rm{O}}}}_{{{\rm{x}}}}}=248.5\ \Omega$, $\frac{\Delta {R}_{{{\rm{Pt}}}}}{{R}_{{{\rm{Pt}}}}}=2.88\times {10}^{-4}$, ${\rho }_{{{\rm{Pt}}}}=60.8\ {{\rm{\mu }}}\Omega \cdot {{\rm{cm}}}$, and ${\theta }_{{{\rm{SH}}}}=0.029$ directly obtain from the YIG/Pt and YIG/Pt/CuO_x_ samples, we estimate the effective orbital Hall angle of CuO_x_ to be ${\theta }_{{{\rm{OH}}},{{\rm{Cu}}}{{{\rm{O}}}}_{{{\rm{x}}}}}=+ 11.8\pm 3.6\%$ which has the same positive sign as the spin Hall angle of Pt. Using the sheet resistance of CuO_x_ ${R}_{{{\rm{Cu}}}{{{\rm{O}}}}_{{{\rm{x}}}}}=248.5\pm 35.7\ \Omega$, we estimate the orbital Hall conductivity in CuO_x_ to be ${\sigma }_{{{\rm{OH}}},{{{\rm{CuO}}}}_{{{\rm{x}}}}}=1187\pm 400\ (\hslash /e)({\Omega }^{-1}{{\rm{cm}}}^{-1})$. These extracted spin and orbital Hall/Nernst angles and conductivities for Pt and CuO_x_ are summarized in Table 1.

**Table 1:** Spin Hall, spin Nernst, orbital Hall, and orbital Nernst angles and conductivities for 3-nm-thick Pt and 2-nm-thick CuO_x_ obtained from this work

	${R}_{S}(\Omega )$	${\theta }_{{{\rm{SH}}}},{\theta }_{{{\rm{OH}}}}(\%)$	${\sigma }_{{{\rm{SH}}}},{\sigma }_{{{\rm{OH}}}}\left((\hslash /e)({\Omega }^{-1}{{\rm{cm}}}^{-1})\right)$	$S({\rm{\mu V/K}})$	${\theta }_{{{\rm{SN}}}},{\theta }_{{{\rm{ON}}}}(\%)$	${\alpha }_{{{\rm{SN}}}},{\alpha }_{{{\rm{ON}}}}\left((\hslash /e)({{\rm{A}}}/{{\rm{m}}}\cdot {{\rm{K}}})\right)$
Pt (3)	202.5 ± 9.1	2.9 ± 0.1	238.5 ± 13.5	+2.24 ± 0.01	−12.5 ± 1.0	−0.23 ± 0.02
CuO_x_ (2)	248.5 ± 35.7	11.8 ± 3.6	1187 ± 400	−4.10 ± 0.61	+22.5 ± 6.8	−0.93 ± 0.34

Error bars represent the propagated uncertainty from the sample length (see Methods).

Notably, as reported in previous studies, the orbital angular momentum generated in CuO_x_ arises primarily from the orbital Rashba–Edelstein effect induced by the Cu-O bonding, with an additional contribution from the orbital Hall effect in the Cu layer^7^. In our analysis, we treat CuO_x_ as an effective single layer; therefore, the extracted effective ${\theta }_{{{\rm{OH}}},{{\rm{Cu}}}{{{\rm{O}}}}_{{{\rm{x}}}}}$ and ${\sigma }_{{{\rm{OH}}},{{\rm{Cu}}}{{{\rm{O}}}}_{{{\rm{x}}}}}$ inherently include both contributions, namely, the interfacial ORE due to the Cu-O hybridization and the OHE originating from Cu.

From these results, we provide compelling evidence that the ONE and OHE induced in CuO_x_ play decisive roles in the additional spin current that reaches the YIG/Pt interface, leading to a pronounced enhancement of the magneto-thermopower and the spin Hall magnetoresistance ratio, respectively.

**Fig. 4:**
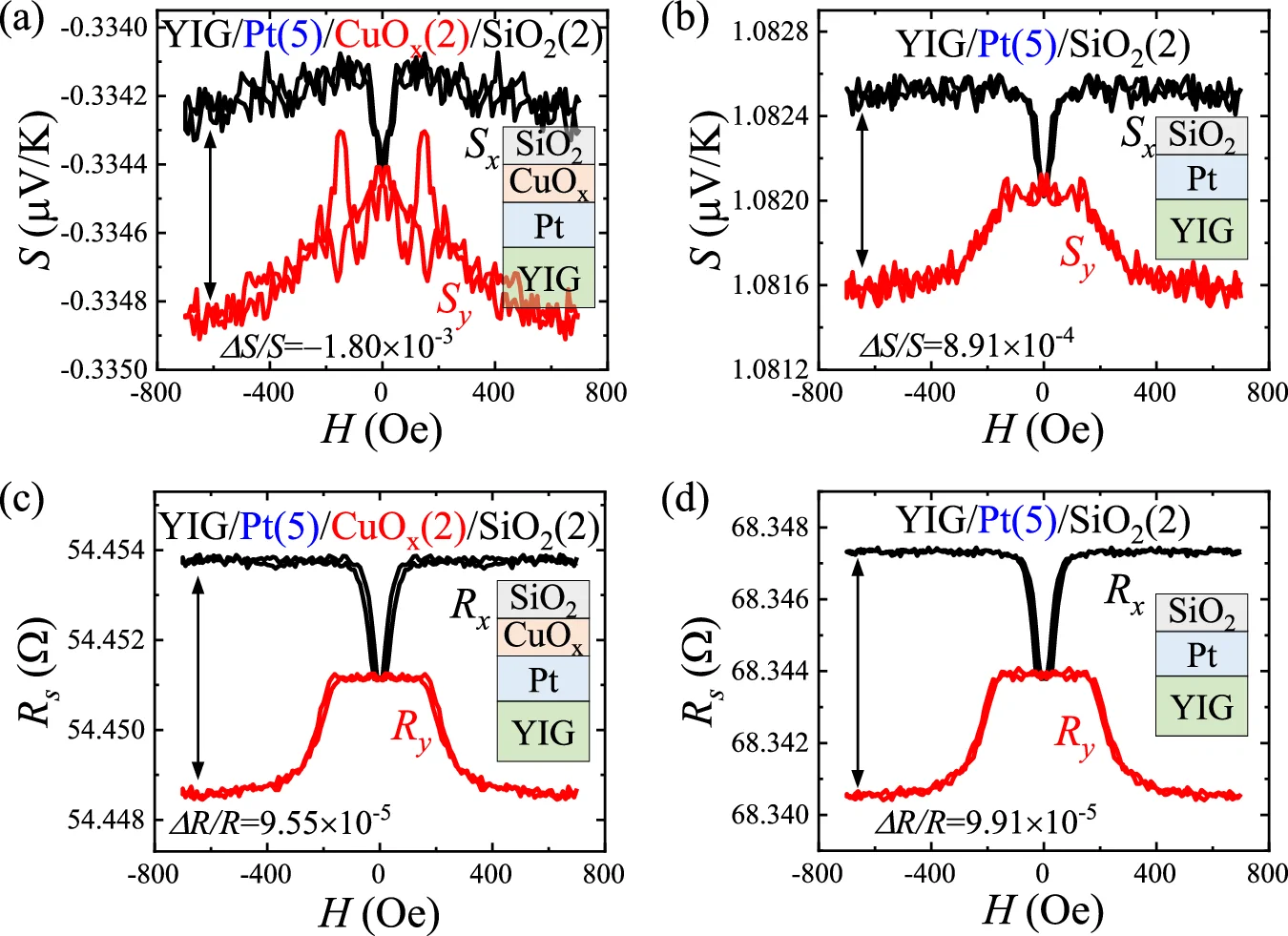
Magneto-thermopower and magnetoresistance measurements for samples with a thicker Pt layer. **a**, **b** Magnetic field-dependent Seebeck coefficient *S* for **a** YIG/Pt(5)/CuO_x_(2)/SiO_2_(2) and **b** YIG/Pt(5)/SiO_2_(2). **c**, **d** Magnetic field-dependent sheet resistance ${R}_{{{\rm{s}}}}$ for **c** YIG/Pt(5)/CuO_x_(2)/SiO_2_(2) and **d** YIG/Pt(5)/SiO_2_(2). Black and red curves represent the values measured with the magnetic field applied along the *x*-axis (${S}_{x}$, ${R}_{x}$) and *y*-axis (${S}_{y}$, ${R}_{y}$), respectively. The magneto-thermopower ratio ΔS/S and magnetoresistance ratio ΔR/R are defined as $({S}_{x}-{S}_{y})/{S}_{x}$ and $({R}_{x}-{R}_{y})/{R}_{x}$, respectively.

We further investigate the thickness dependence of the magneto-thermopower and magnetoresistance ratio using a thicker Pt layer by preparing the YIG/Pt(5)/CuO_x_(2) and YIG/Pt(5) samples. As shown in the magneto-thermopower (Fig. 4a, b) and the magnetoresistance (Fig. 4c, d) measurements, the Seebeck voltage changes sign for samples with and without CuO_x_, due to the opposite Seebeck coefficients of CuO_x_ and Pt. The same negative ${S}_{{{\rm{Cu}}}{{{\rm{O}}}}_{{{\rm{x}}}}}$ for the 2-nm-thick CuO_x_ is consistent with that observed in Fig. 2b. The effective magneto-thermopower and magnetoresistance ratio for Pt in the YIG/Pt(5)/CuO_x_(2) sample are estimated to be $\frac{\Delta {S}_{{{\rm{Pt}}}}}{{S}_{{{\rm{Pt}}}}} \sim \left(8.54\pm 0.84\right)\times {10}^{-4}$ and $\frac{\Delta {R}_{{{\rm{Pt}}}}}{{R}_{{{\rm{Pt}}}}} \sim \left(1.20\pm 0.15\right)\times {10}^{-4}$, respectively. These values are comparable to those directly obtained from the YIG/Pt(5) sample with $\frac{\Delta {S}_{{{\rm{Pt}}}}}{{S}_{{{\rm{Pt}}}}} \sim \left(8.91\pm 0.64\right)\times {10}^{-4}$ and $\frac{\Delta {R}_{{{\rm{Pt}}}}}{{R}_{{{\rm{Pt}}}}} \sim (0.99\pm 0.45)\times {10}^{-4}$. These results show that with a thicker Pt layer, the additional spin current, induced from the SOC-mediated orbital current in CuO_x,_ that can reach the YIG/Pt interface, is significantly reduced. This behavior supports our assumption that orbital-to-spin conversion occurs immediately at the Pt/CuO_x_ interface, and decays rapidly within the Pt layer.

**Fig. 5:**
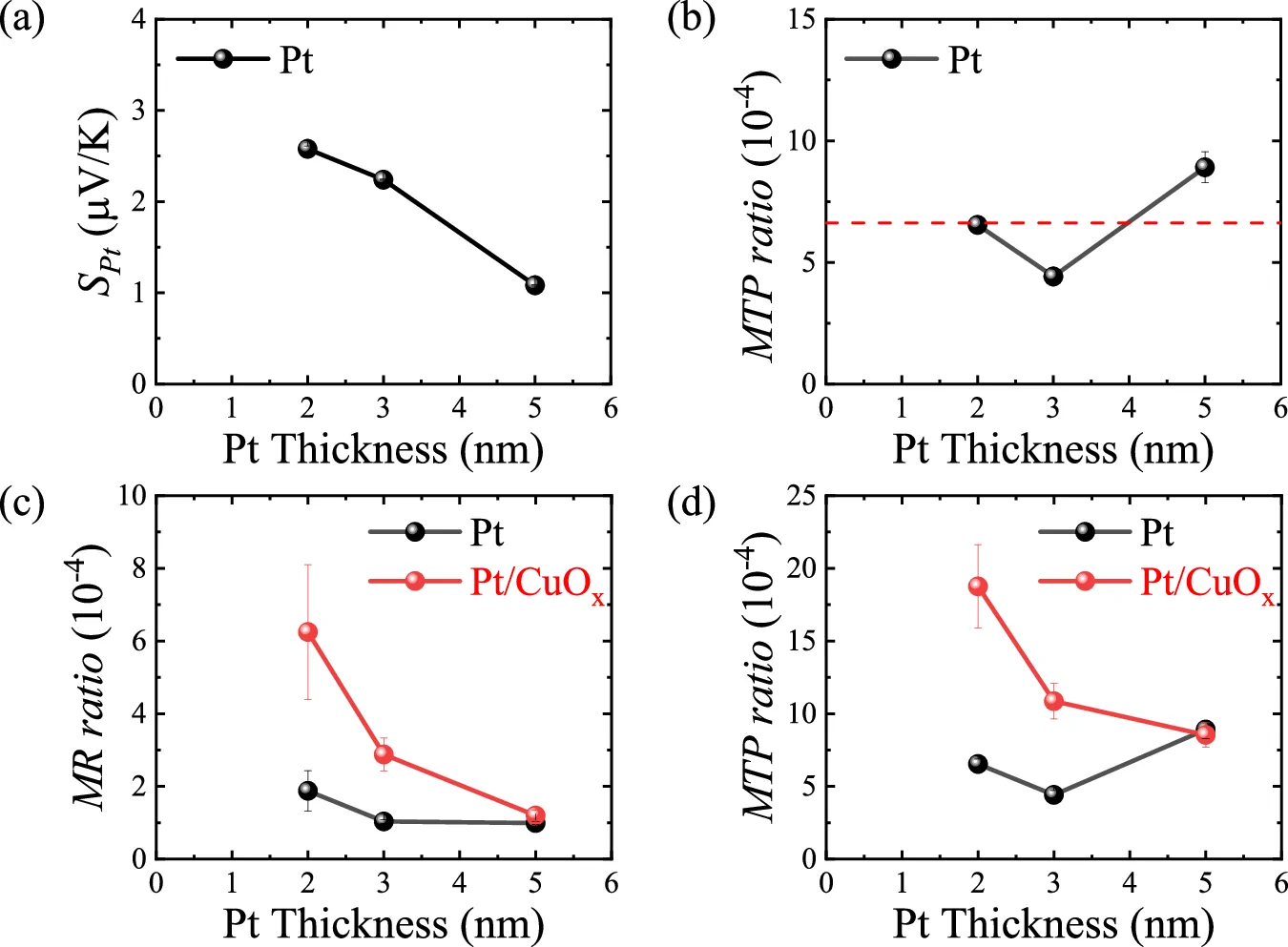
Pt-thickness dependence of the thermoelectric and electrical signals. **a** Pt-thickness dependence of the Seebeck coefficient (${S}_{{{\rm{Pt}}}}$) and **b** magneto-thermopower (MTP) ratio for the YIG/Pt(*t*)/SiO_2_(2) sample. The red dashed line in (**b**) indicates the average YIG/Pt MTP ratio across the measured thickness range, highlighting its weak thickness dependence compared to the Seebeck coefficient in (**a**). **c**, **d** Thickness-dependent effective **c** magnetoresistance (MR) ratio and **d** MTP ratio for Pt in YIG/Pt(*t*)/SiO_2_(2) (black) and YIG/Pt(*t*)/CuO_x_(2)/SiO_2_(2) (red) samples. Error bars represent the propagated uncertainty from the sample length.

The systematic comparison of Pt-thickness-dependent MR and MTP ratios between YIG/Pt(*t*) and YIG/Pt(*t*)/CuO_x_(2) samples, with *t* ranging from 2 to 5 nm, is shown in Fig. 5. For the YIG/Pt(*t*) samples, the Seebeck coefficient of Pt varies substantially with thickness, as shown in Fig. 5a. The result confirms the strong thickness dependence of the Seebeck coefficient for ultrathin films. In contrast, the magneto-thermopower ratio exhibits a much weaker thickness dependence and shows no corresponding enhancement in the thin-Pt regime, as shown in Fig. 5b.

In Fig. 5c, d, we compare the effective thickness-dependent MR and MTP ratios of the Pt layer, for the YIG/Pt(*t*) and YIG/Pt(*t*)/CuO_x_(2) samples, represented by the black and red symbols, respectively. For the samples containing CuO_x_(2), both the MR and MTP ratios increase markedly as the Pt thickness is reduced from 5 to 2 nm, whereas the corresponding thickness dependence in the YIG/Pt(*t*) samples is considerably weaker. This contrasting behavior is consistent with that reported in ref. ^6^, where the signals of CuO_x_-capped and uncapped samples diverge in the thin-Pt regime and gradually converge as the Pt thickness increases. Taken together, the parallel enhancement of the MR and MTP ratios in the thin-Pt regime for the YIG/Pt(*t*)/CuO_x_(2) samples provides additional transport evidence supporting orbital current generation in CuO_x_.

In summary, we report the first transport evidence for the orbital Nernst effect in YIG/Pt/CuO_x_ heterostructures. Magneto-thermopower measurements reveal a magneto-thermopower contribution consistent with a thermally generated orbital current in CuO_x_, characterized by a large positive orbital Nernst angle and a negative orbital Nernst conductivity that is up to six times larger than the spin Nernst conductivity in Pt. Complementary magnetoresistance measurements further confirm a robust orbital Hall effect in CuO_x_, with a significant orbital Hall angle and conductivity that are up to an order of magnitude larger than the spin Hall conductivity in Pt. The absence of both magnetoresistance and magneto-thermopower signals in YIG/CuO_x_ samples confirms that CuO_x_ alone does not generate a spin current. Moreover, the systematic Pt-thickness dependence of both the MR and MTP ratios indicates that orbital-to-spin conversion occurs predominantly at the Pt/CuO_x_ interfaces and decays rapidly within Pt. From these measurements, we extract a large orbital Hall angle ${\theta }_{{{\rm{OH}}},{{\rm{Cu}}}{{{\rm{O}}}}_{{{\rm{x}}}}}=+ 11.8\pm 3.6\%$ and a substantial orbital Nernst angle of ${\theta }_{{{\rm{ON}}},{{\rm{Cu}}}{{{\rm{O}}}}_{{{\rm{x}}}}}=+ 22.5\pm 6.8\%$ for CuO_x_. By providing transport evidence for the orbital Nernst effect, our work supports that the orbital degree of freedom can be utilized as a powerful and previously unexplored pathway for thermoelectric conversion, opening new opportunities for heat-energy harvesting based on orbital caloritronics.

## Methods

### Sample fabrication

The polycrystalline yttrium iron garnet (Y_3_Fe_5_O_12_, YIG) slabs with widths of 2-2.5 mm, lengths of 9-11 mm, and thicknesses of 0.5 mm are used as substrates. A series of heterostructures in the form of YIG/X/SiO_2_ is studied, where X denotes Pt, Pt/CuO_x_, and CuO_x_ thin layers. The transition metal layers are deposited on YIG substrates by DC magnetron sputtering. For the CuO_x_ layer, a Cu layer is first deposited and then naturally oxidized in air for 2 days. The capping SiO_2_ layer is deposited by RF sputtering.

### Measurement

For both the magnetoresistance (MR) and magneto-thermopower (MTP) measurements, the voltage is obtained using a Keithley 2182A nanovoltmeter under an applied in-plane magnetic field. The background noise for the voltage is ~30 nV during one field sweep. To suppress noise, at each magnetic-field step, ten voltage readings are recorded and averaged, reducing the noise level by approximately one order of magnitude. Moreover, each measurement is repeated over 50 cycles. This procedure ensures reliable and reproducible data while maintaining high sensitivity. Each full measurement cycle requires approximately one day per sweep direction, enabling the detection of spin Nernst magneto-thermopower signals with an ultralow signal-to-noise ratio.

### Error analysis

The uncertainty in the current-carrying length arises from the finite width (~1 mm) of the indium contacts. For a nominal sample length of 10 mm, this corresponds to an effective length of 9 ± 1 mm. This geometric uncertainty is propagated into the calculated transport quantities and is presented as the error bars in the figures.

## Supplementary information

Supplementary Information

Transparent Peer Review file

## Data Availability

The data that support the findings of this study are available from the corresponding author upon reasonable request.
