# Supplementary material for: Transport evidence for the orbital Nernst effect
Source: Nat Commun. 2026 Sep 22;17:11032. doi: 10.1038/s41467-026-77878-2 (PMC13686958; doi:10.1038/s41467-026-77878-2)
Supplement: Supplementary file 1 [file 41467_2026_77878_MOESM1_ESM.pdf]

Supplementary Information for

Transport Evidence for the Orbital Nernst Effect

Wen-Yuan Li[1, 2], Chun-Chi Chang[1], Ssu-Yen Huang[1, 3] and Danru Qu[2, 3*]

[1]*Department of Physics, National Taiwan University, Taipei 10617, Taiwan*

[2]*Center for Condensed Matter Sciences, National Taiwan University, Taipei 10617, Taiwan*

[3]*Center of Atomic Initiatives for New Materials, National Taiwan University, Taipei 10617, Taiwan*

[*]Corresponding author. danru@ntu.edu.tw (D.Q.)

Supplementary Note 1. Details of the magneto-thermopower and magnetoresistance measurements.

For the magneto-thermopower measurement, as illustrated in Fig. S1, the two ends of the sample are mechanically clamped: one end is thermally anchored to a heater, while the other end is connected to an aluminum-based heat sink. The heat flow is generated by applying a voltage to the heater using a Keithley 2231A source-measurement unit. The temperatures at the two ends of the sample are measured using thermocouples connected to a Keithley 2182A thermometer. The thermoelectric voltage is measured using another Keithley 2182A nanovoltmeter with Cu connecting wires. Importantly, both the thermocouples and the voltage leads are attached to the same positions on the sample, ensuring that the effective lengths for voltage and temperature measurements are identical. For each heating condition, both the

voltages and temperatures are recorded after stabilization for five minutes following the setting of the heater voltage.

The magneto-thermopower measurement is performed by fixing the heating power and recording the Seebeck voltage while sweeping the external magnetic field. The magnetic field is swept from $+700$ Oe to -700 Oe and back to $+700$ Oe in steps of 10 Oe. The background noise level for the voltage is approximately 30 nV during the field sweep. To suppress noise, at each magnetic-field step, ten voltage readings are recorded and averaged, reducing the noise level by approximately one order of magnitude. Moreover, each measurement is repeated over 50 cycles. This procedure ensures reliable and reproducible data while maintaining high sensitivity. Each full measurement cycle requires approximately one day per sweep direction, enabling the detection of spin Nernst magneto-thermopower signals with an ultralow signal-to-noise ratio.

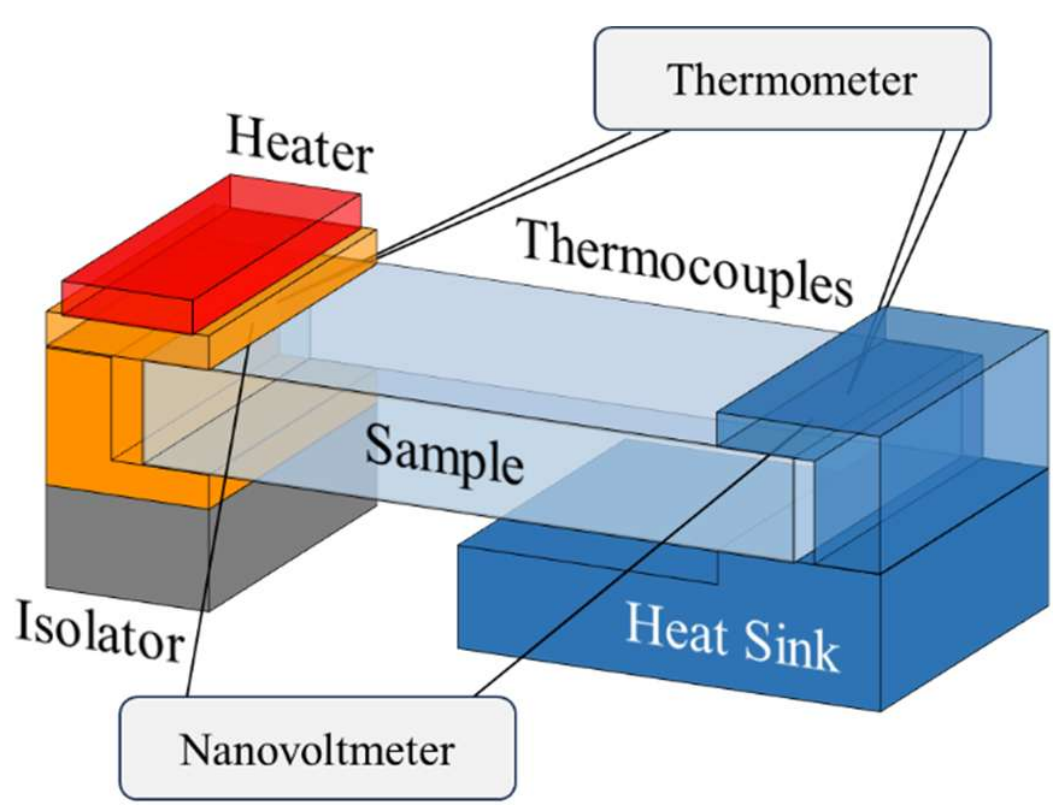

Fig. S1. Schematic illustration of the thermal measurement setup.

For the magnetoresistance (MR) measurement of the thin film, the sample is electrically connected to the nanovoltmeters and current sources via Cu wires. To ensure a uniform charge-current distribution across the film, an indium strip is used to contact the film surface, as illustrated in Fig. S2. This configuration minimizes current crowding and provides stable, low-resistance electrical contacts.

Because the indium contact has a finite width, it introduces an uncertainty in the effective current path, which in turn leads to an uncertainty in the determination of the sheet resistance and resistivity. In our measurements, this geometric uncertainty associated with the contact width constitutes the dominant source of error in the resistance determination.

In the present samples, the total length of the film is approximately 10 mm, whereas the width of the indium contact is about 0.5 mm. As a result, the relative uncertainty introduced by the contact geometry is small, and its influence on the extracted MR values is limited. Consequently, the MR data reported in this work are considered reliable within the estimated error bars.

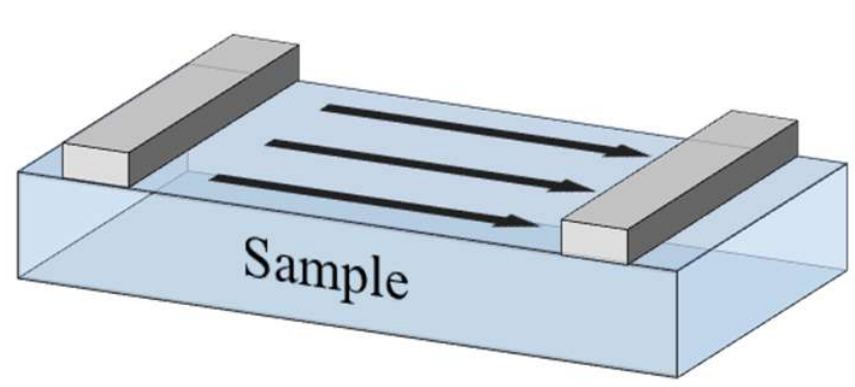

Fig. S2. Schematic illustration of the electrical measurement setup.

Supplementary Note 2. Separation of voltage signals induced by the in-plane heat current.

Although in our study, we intend to supply an in-plane heat current (∇T_x) by placing a heater and a heat sink at both sides of the sample, as shown in Fig. S3(a), an out-of-plane temperature gradient (∇T_z) is unavoidable and cannot be completely eliminated due to different thermal conductivities for the film and substrate. Under a finite $\nabla_z T$, a longitudinal magnonic spin current is generated in the ferromagnetic insulator yttrium iron garnet (YIG) through the spin Seebeck effect (SSE) [1, 2]. In a ferromagnetic-insulator/nonmagnetic metal (FMI/NM) bilayer, this spin current can be converted into an electrical voltage via the inverse spin Hall effect (ISHE), resulting in an SSE/ISHE contribution in addition to the spin Nernst magneto-thermopower (SNMT) signal. As shown in Fig. S3(b) and (e), the thermal voltages for both the YIG/Pt/CuOx and YIG/Pt samples are dominated by the asymmetric contributions induced by the SSE/ISHE.

Crucially, the SSE and SNMT contributions exhibit different symmetries with respect to the magnetic field. The SSE voltage is antisymmetric in the magnetic field, whereas the SNMT voltage is symmetric. This symmetry distinction enables a reliable separation of the two contributions. The symmetric (Fig. S3(d) and (g)) and antisymmetric (Fig. S3(c) and (f)) components of the measured voltage are obtained by applying the following operations:

$$V_{\rm sym}(H) = \frac{V(+H) + V(-H)}{2} \tag{S1}$$

$$V_{\rm asym}(H) = \frac{V(+H) - V(-H)}{2} \tag{S2}$$

where $V_{\rm sym}(H)$ and $V_{\rm asym}(H)$ correspond to the symmetric SNMT and antisymmetric SSE components, respectively. By sweeping the magnetic field from positive to negative values and back, a complete voltage hysteresis loop containing both forward and backward field scans is measured, allowing the two contributions to be separated unambiguously.

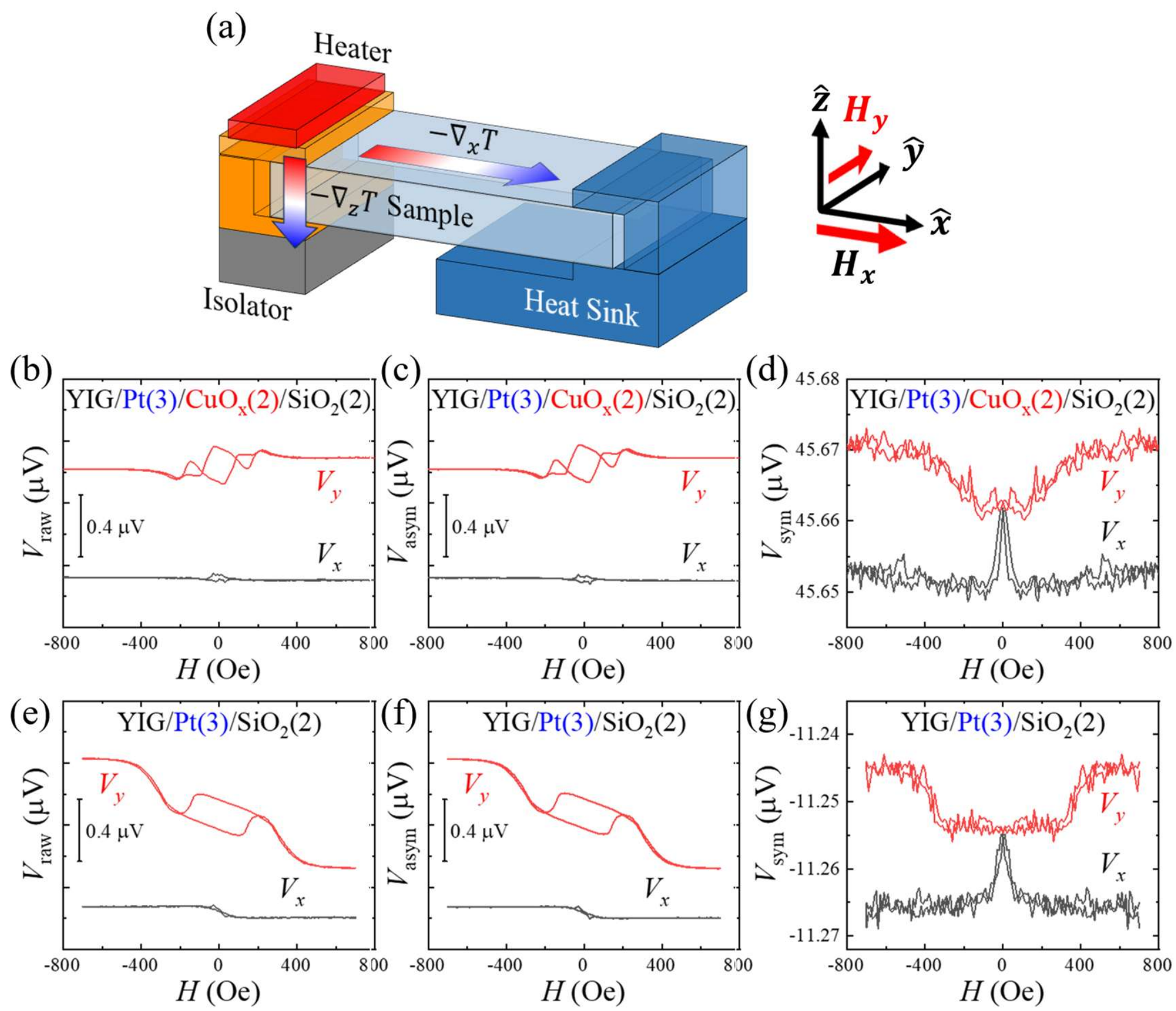

Fig. S3. (a) Schematic illustration of the measurement setup. (b) Unsymmetrized, (c) asymmetrized, and (d) symmetrized thermal voltages for YIG/Pt(3)/CuO_x(2)/SiO_2(2). (e) Unsymmetrized, (f) asymmetrized, and (g) symmetrized thermal voltages for

YIG/Pt(3)/SiO_2(2).

Supplementary Note 3. Magnetization for the polycrystalline YIG slab.

We measure the magnetic hysteresis loops of a YIG slab using vibrating sample magnetometry (VSM), with the magnetic field applied along the *x* and *y* directions. The YIG slab has dimensions of 12 mm × 3 mm, with the long axis along *x*.

The VSM results, as shown in Fig. S4(a), reveal that the saturation field H_x is smaller than H_y, indicating that the magnetization aligns more easily along the long axis due to shape anisotropy. This behavior is consistent with the hysteretic responses observed in the SMR and SNMT measurements under H_x and H_y, as shown in Fig. S4 (b)-(e).

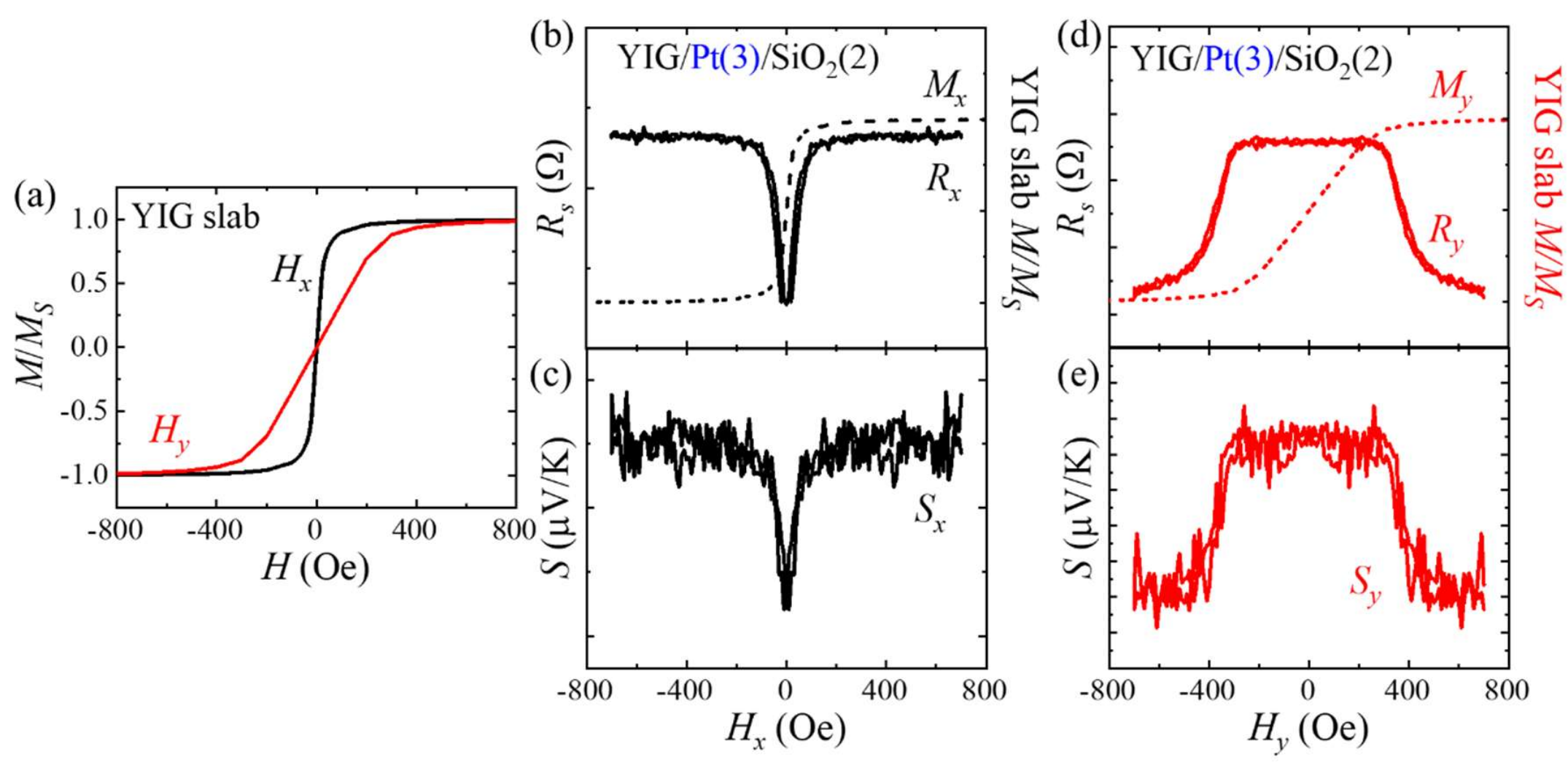

Fig. S4. Vibrating sample magnetometry (VSM) measurements for the YIG slab with the magnetic field applied along the *x*-axis (black curve) and *y*-axis (red curve). Similar saturation behavior is observed in the (b), (d) magnetoresistance and (c), (e) magneto-thermopower measurements for the YIG/Pt/SiO_2 samples and other YIG-slab-based samples used in the study. The dotted curves in (b) and (d) correspond to the magnetization curves shown in (a).

Supplementary Note 4. Seebeck coefficient of the film.

The Seebeck coefficient S is determined by applying different levels of heat flow and measuring the corresponding temperature difference (ΔT) and thermal voltage (ΔV) between the two ends of the sample. Since the voltage and temperature terminals are the same, we have

$$S = \frac{E}{\nabla T} = -\frac{\nabla V}{\nabla T} = -\frac{\Delta V}{\Delta T} \tag{S1}$$

By fitting the linear relation between the measured voltage and temperature difference, we obtain an effective Seebeck coefficient S' for the film. However, since the bonding Cu wires themselves possess a finite Seebeck coefficient, they introduce additional thermoelectric voltages that must be accounted for.

As illustrated in Fig. S5(a), when a temperature gradient is applied from left to right (indicated by the French-flag arrow), a Seebeck voltage develops in the sample, given by $V_{\mathrm{film}}^{+} - V_{\mathrm{film}}^{-}$, under a temperature difference $T_{\mathrm{H}} - T_{\mathrm{L}}$. Meanwhile, the voltmeter is maintained at the ambient temperature T_{env}, which is different from both T_{H} and T_{L}, leading to additional thermoelectric contributions from the connecting wires. As a result, the voltage measured by the voltmeter is the sum of the Seebeck voltages generated in the sample and in the two wires:

$$V^{+} - V^{-} = (V^{+} - V_{\mathrm{film}}^{+}) + (V_{\mathrm{film}}^{+} - V_{\mathrm{film}}^{-}) + (V_{\mathrm{film}}^{-} - V^{-}) \tag{S3}$$

wire env film wire env

$$= -(S_{\mathrm{film}} - S_{\mathrm{wire}})(T_{\mathrm{H}} - T_{\mathrm{C}})$$

Accordingly, the Seebeck coefficient of the film is given by

$$S_{\mathrm{film}} = S' + S_{\mathrm{wire}} \tag{S4}$$

where the effective Seebeck coefficient S' directly obtained from the voltmeter is defined as $S' = -(V^{+} - V^{-})/(T_{\mathrm{H}} - T_{\mathrm{C}})$.

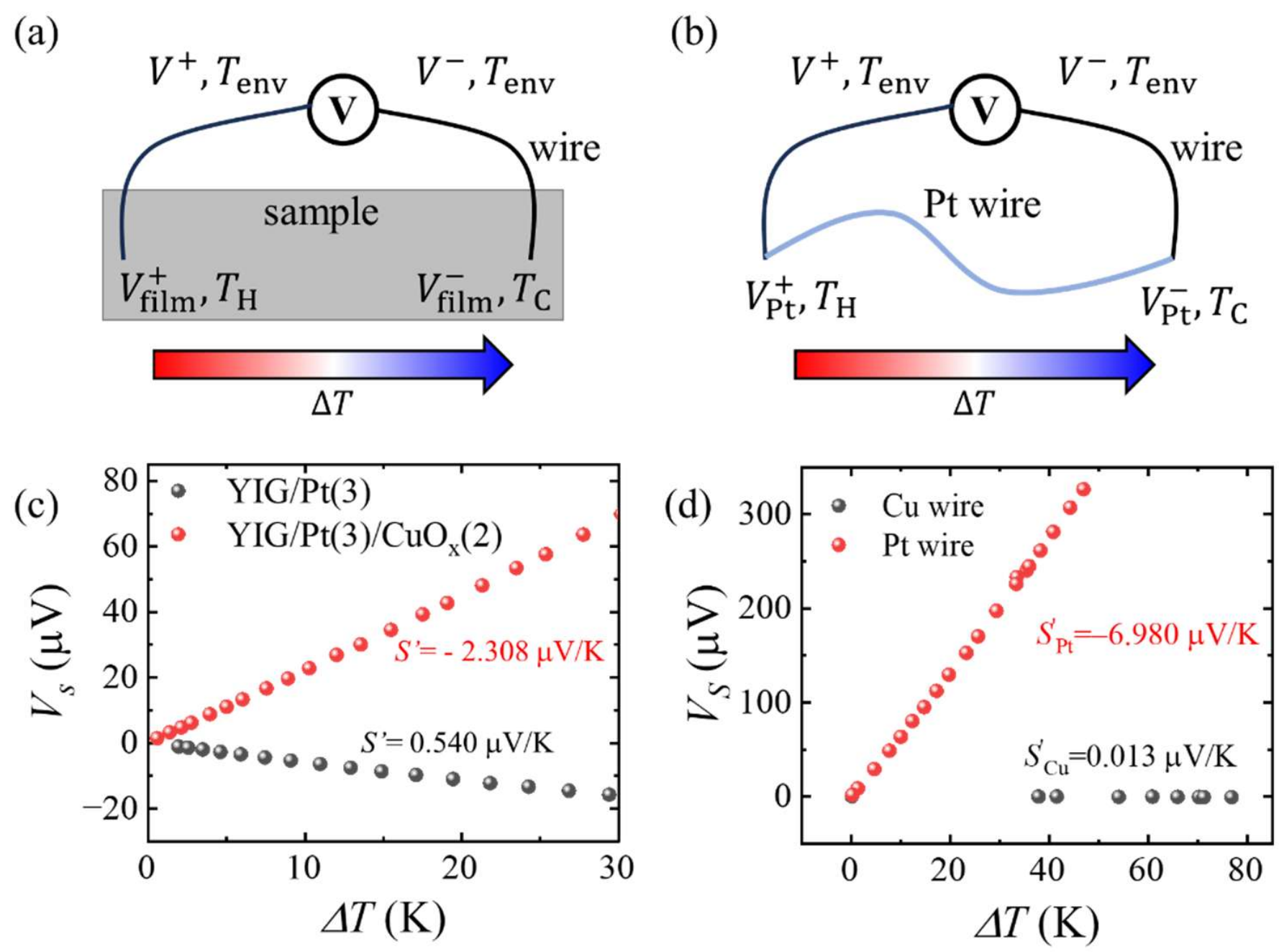

Fig. S5. Schematic illustrations of the local temperature and voltage for (a) the sample, (b) the Pt wire, with the bonding Cu wires. The effective Seebeck coefficient for the (c) YIG/Pt and YIG/Pt/CuO$_x$ samples, and (d) Pt and Cu wires before subtracting the contributions from the bonding Cu wires.

To determine S_{wire}, we calibrate the setup using materials with well-established Seebeck coefficients. As shown in Fig. S5(b), the sample is replaced by a Pt wire with a known

Seebeck coefficient of $S_{\mathrm{Pt}} = -5.3\ \mu\mathrm{V/K}$ [3]. By applying a current through the heater, a temperature difference ΔT is created across the Pt wire and measured using thermocouples. From the result in Fig. S5(d), the total Seebeck coefficient for Pt wire obtained directly from the voltmeter is $S'_{\mathrm{Pt}} = -6.98\ \mu\mathrm{V/K}$. The difference between S'_{Pt} and S_{Pt} confirms the additional contribution from the connecting wires. Substituting these values into Eq. S4, we obtain the Seebeck coefficient of the Cu wire as $S_{\mathrm{wire}} = 1.68\ \mu\mathrm{V/K}$. A similar calibration procedure using a Cu wire yields a total Seebeck coefficient of $S'_{\mathrm{Cu}} = 0.013\ \mu\mathrm{V/K}$, confirming that the contribution from Cu bonding wires must be considered to obtain the correct Seebeck coefficient for the sample.

Fig. S5(c) shows the dependence of the thermoelectric voltage on the temperature difference for the YIG/Pt (black dots) and YIG/Pt/CuO_x (red dots) samples. Both samples exhibit a clear linear dependence. From linear fitting, the effective Seebeck coefficients are determined to be $S' = 0.540\ \mu\mathrm{V/K}$ and $-2.308\ \mu\mathrm{V/K}$ for the YIG/Pt and YIG/Pt/CuOx samples, respectively. After subtracting the Cu bonding wire contributions, the intrinsic Seebeck coefficients for the YIG/Pt and YIG/Pt/CuO_x samples are obtained as $S = 2.24\ \mu\mathrm{V/K}$ and $-0.61\ \mu\mathrm{V/K}$, respectively.

Supplementary Note 5. Estimation of the effective magnetoresistance and magneto-thermopower ratio for the Pt layer in YIG/Pt/CuO_x.

In our experiments, we conduct both the magnetoresistance (MR) and magneto-thermopower (MTP) measurements on both the YIG/Pt and YIG/Pt/CuO_x samples. Since both the MR and MTP ratios originate from the reflection and absorption of spin currents at the YIG/Pt interfaces, we estimate the effective MR and MTP ratios for Pt while treating the CuO_x layer as a shunt layer. Under this assumption, the SOC-mediated orbital current contribution from CuO_x is included as the enhancement of the effective MR and MTP ratios in Pt. This assumption also means that only the resistance of Pt changes with the magnetic field, but not the CuO_x.

For the YIG/Pt sample, the sheet resistance of is obtained as $R_{\mathrm{Pt}} = 202.5\ \Omega$. For the YIG/Pt/$CuO_x$ sample, with the additional CuO_x layer, the sheet resistance is reduced to $R_{tot} = 111.6\ \Omega$. From the parallel resistance model, for the YIG/Pt/CuO_x sample, we have

$$\frac{1}{R_{tot}} = \frac{1}{R_{\mathrm{Pt}}} + \frac{1}{R_{\mathrm{CuO_x}}} \tag{S5}$$

in which both the sheet resistance for the whole sample (R_{tot}) and the sheet resistance for the Pt layer (R_{Pt}) vary under H_x and H_y, but not the sheet resistance for the CuO_x layer ($R_{\mathrm{CuO_x}}$). Therefore, we have

$$\frac{1}{R_{tot} + \Delta R_{tot}} - \frac{1}{R_{tot}} = \frac{1}{R_{\mathrm{Pt}} + \Delta R_{\mathrm{Pt}}} - \frac{1}{R_{\mathrm{Pt}}} \tag{S6}$$

where ΔR denotes the resistance change under H_x and H_y. We can rewrite Eq. S6 as

$$\Delta R_{\mathrm{Pt}} = \frac{R_{\mathrm{Pt}}^2 \Delta R_{tot}}{R_{tot}^2 + (R_{\mathrm{Pt}} - R_{tot})\Delta R_{tot}} \tag{S7}$$

which is the effective resistance difference of the Pt layer in the YIG/Pt/CuO_x sample. Note that YIG is an insulator with no current flow through.

For the MTP measurement, we also use the parallel-resistance model to estimate the Seebeck coefficient for each layer

$$\frac{S_{tot}}{R_{tot}} = \frac{S_{\mathrm{Pt}}}{R_{\mathrm{Pt}}} + \frac{S_{\mathrm{CuO_x}}}{R_{\mathrm{CuO_x}}} \tag{S8}$$

Under the same assumption that CuO_x functions as a shunt layer, we estimate the effective Seebeck coefficient change for Pt

$$\frac{S_{tot} + \Delta S_{tot}}{R_{tot} + \Delta R_{tot}} - \frac{S_{tot}}{R_{tot}} = \frac{S_{\mathrm{Pt}} + \Delta S_{\mathrm{Pt}}}{R_{\mathrm{Pt}} + \Delta R_{\mathrm{Pt}}} - \frac{S_{\mathrm{Pt}}}{R_{\mathrm{Pt}}} \tag{S9}$$

where ΔS denotes the change of Seebeck coefficient under H_x and H_y. We can rewrite Eq. S9 as

$$\Delta S_{\mathrm{Pt}} = \frac{R_{\mathrm{Pt}}}{R_{tot}^2 + (R_{\mathrm{Pt}} - R_{tot})\Delta R_{tot}} \left(R_{tot}\Delta S_{tot} + (S_{\mathrm{Pt}} - S_{tot})\Delta R_{tot}\right) \tag{S10}$$

which is the Seebeck coefficient difference of the Pt layer in YIG/Pt/CuO_x sample under H_x and H_y.

Supplementary Note 6. The simplified drift-diffusion model.

To establish the relationship between the experimentally measured electrical and thermal signals and the spin Hall, spin Nernst, orbital Hall, and orbital Nernst angles, we extend the drift-diffusion formalism developed for magnetoresistance [4] and magneto-thermopower [1] for the ferromagnetic insulator/ non-magnetic metal bilayer as shown in Fig. S6(a), to a three-layer system that consists of YIG, Pt, and CuO_x layers, as shown in Fig. S6(b). In the model, the ferromagnetic insulator YIG provides a spin-selective boundary condition, while the Pt layer serves as both the generator and detector of spin current via the spin Hall, spin Nernst, and inverse spin Hall effects. The CuO_x layer provides an additional source of orbital current, which is converted into spin current upon entering the Pt layer. Since both spin transport and charge/heat-spin interconversion mainly occur inside the Pt layer, the following analysis is restricted to Pt. The role of YIG and CuO_x is incorporated through boundary conditions for the spin current at the Pt/YIG and Pt/CuO_x interfaces.

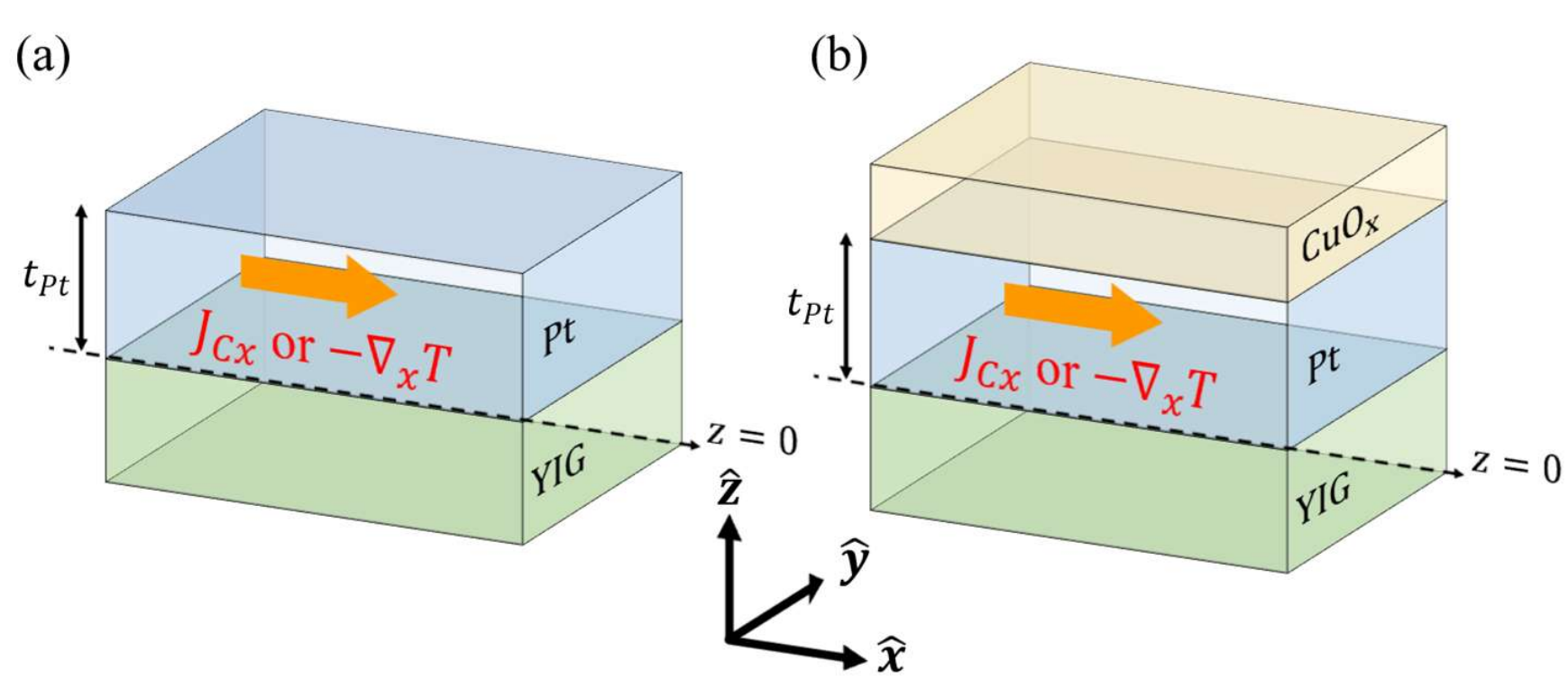

Fig. S6. Schematic illustrations of the simplified drift diffusion model for the (a) YIG/Pt and

(b) YIG/Pt/CuO_x samples.

In particular, by replacing the Pt/vacuum interface with an additional spin-current source at the Pt/CuO_x interface, the distribution of spin accumulation in Pt is modified, resulting in a distinct inverse spin Hall signal. Analysis of this signal allows us to extract the magnitude of the injected spin-orbital current from the CuO_x layer.

In our experimental geometry, as shown in Fig. S6(b), both the charge current and the heat current flow along the *x*-axis, the spin accumulation develops along the *y*-axis, the corresponding spin current propagates along the *z*-axis, and the resistance or Seebeck voltage is measured along the *x*-axis. Under these constraints, only j_{Cx}, j_{qx}, and j_{Sz}^{y} are non-zero. Therefore, the Onsager relation is reduced to a compact 3×3 form:

$$\begin{pmatrix} j_{Cx} \\ j_{qx} \\ j_{Sz}^{y} \end{pmatrix} = \sigma \begin{pmatrix} 1 & S & -\theta_{\mathrm{SH}} \\ S & L_{22}T & -S\theta_{\mathrm{SN}} \\ \theta_{\mathrm{SH}} & S\theta_{\mathrm{SN}} & 1 \end{pmatrix} \begin{pmatrix} E_{\mathrm{eff}} \\ -\partial_x T \\ -\partial_z \mu_S^{y}/2e \end{pmatrix} \tag{S11}$$

Here, j_{Cx} is the electric current density, j_{qx} is the energy flow, and j_{Sz}^{y} is the spin current which flows along the *z*-axis with spin polarization along the *y*-axis. For the right-hand side of the equation, σ is the electrical conductivity, S is the Seebeck coefficient, θ_{SH} and θ_{SN} is the spin Hall and spin Nernst angle, L_{22} is a parameter which relates to the thermal conductivity, $E_{\mathrm{eff}} = E_{\mathrm{ext}} + \partial_x \mu_0/e$ is the effective electric field which include the drift and diffusion electromotive

force, μ_0 is the electrochemical potential, and μ_S^y is the spin dependent electrochemical potential under *y* polarization, which relates to the spin accumulation as $\mu_S = \mu_S^y - \mu_0$.

In the steady state, the spin accumulation in Pt satisfies the one-dimensional spin drift-diffusion equation

$$\partial_z^2 \mu_S = \frac{\mu_S}{\lambda_S^2} \tag{S12}$$

where λ_S is the spin diffusion length of Pt. The general solution is written as

$$\mu_S(z) = A e^{-z/\lambda_S} + B e^{z/\lambda_S} \tag{S13}$$

with coefficients A and B determined by the boundary conditions. The diffusing spin current is determined by the gradient of the spin accumulation

$$j_S^{\mathrm{diff}} = -\frac{\sigma_{\mathrm{Pt}}}{2e} \partial_z \mu_S \tag{S14}$$

Calculation of the magnetoresistance (MR)

For the calculation of the MR, we have $\nabla T = 0$, a charge current $j_C = \sigma_{\mathrm{Pt}}(E + \theta_{\mathrm{SH}} \partial_z \mu_S / 2e)$ and the spin current $j_{Sz}^y = \sigma_{\mathrm{Pt}}(\theta_{\mathrm{SH}} E - \partial_z \mu_S / 2e)$. As shown in Fig. S6(b), the boundary condition for the spin current at the YIG/Pt interface, where $z = 0$, depends on the magnetization (m) direction of YIG. For $m \parallel \hat{x}$, we have $j_{Sz}^y(z = 0) = -\frac{G_r}{e} \mu_S(z = 0)$, where G_r is the spin mixing conductance at YIG/Pt interface. For $m \parallel \hat{y}$, we have $j_{Sz}^y(z = 0) = 0$. On

the other hand, the spin current at Pt/CuO_x interface, $j_{Sz}^{y}(z=t)=\theta_{\mathrm{OH}}\sigma_{\mathrm{CuO_x}}E$, does not depend on the magnetization. Using these boundary condition, solving A and B factors, we could obtain the spin accumulation

$$\mu_S(z)=-\frac{2eE\lambda_S}{\sigma_{\mathrm{Pt}}\sinh\frac{t}{\lambda_S}}\left[\sigma_{\mathrm{CuO_x}}\theta_{\mathrm{OH}}\cosh\frac{z}{\lambda_S}+\sigma_{\mathrm{Pt}}\theta_{\mathrm{Pt}}\left(\cosh\frac{t-z}{\lambda_S}-\cosh\frac{z}{\lambda_S}\right)\right.$$
$$\left.-\,m\times(m\times\hat{y})\left(\sigma_{\mathrm{CuO_x}}\theta_{\mathrm{OH}}\operatorname{csch}\frac{t}{\lambda_S}+\sigma_{\mathrm{Pt}}\theta_{\mathrm{SH}}\tanh\frac{t}{2\lambda_S}\right)\frac{2\lambda_S G_{\mathrm{r}}\cosh\frac{t-z}{\lambda_S}}{\sigma_{\mathrm{Pt}}+2\lambda_S G_{\mathrm{r}}\coth\frac{t}{\lambda_S}}\right] \tag{S15}$$

spin current

$$j_{Sz}^{y}(z)=\frac{2E}{\sinh\frac{t}{\lambda_S}}\left[\sinh\frac{z}{2\lambda_S}\left(\sigma_{\mathrm{CuO_x}}\theta_{\mathrm{OH}}\cosh\frac{z}{2\lambda_S}+\sigma_{\mathrm{Pt}}\theta_{\mathrm{SH}}\left(\cosh\frac{2t-z}{2\lambda_S}-\cosh\frac{z}{2\lambda_S}\right)\right)\right.$$
$$\left.+\,m\times(m\times\hat{y})\left(\sigma_{\mathrm{CuO_x}}\theta_{\mathrm{OH}}\operatorname{csch}\frac{t}{\lambda_S}+\sigma_{\mathrm{Pt}}\theta_{\mathrm{SH}}\tanh\frac{t}{2\lambda_S}\right)\frac{\lambda_S G_{\mathrm{r}}\sinh\frac{t-z}{\lambda_S}}{\sigma_{\mathrm{Pt}}+2\lambda_S G_{\mathrm{r}}\coth\frac{t}{\lambda_S}}\right] \tag{S16}$$

and the charge current density

$$j_C(z)=\sigma_{\mathrm{Pt}}E+\frac{E}{\sinh\frac{t}{\lambda_S}}\left[-\sigma_{\mathrm{CuO_x}}\theta_{\mathrm{OH}}\theta_{\mathrm{SH}}\sinh\frac{z}{\lambda_S}+\sigma_{\mathrm{Pt}}\theta_{\mathrm{SH}}^{2}\left(\sinh\frac{t-z}{\lambda_S}+\sinh\frac{z}{\lambda_S}\right)\right.$$
$$\left.-\left(1-m_y^2\right)\left(\sigma_{\mathrm{CuO_x}}\theta_{\mathrm{OH}}\operatorname{csch}\frac{t}{\lambda_S}+\sigma_{\mathrm{Pt}}\theta_{\mathrm{SH}}\tanh\frac{t}{2\lambda_S}\right)\theta_{\mathrm{SH}}\frac{2\lambda_S G_{\mathrm{r}}\sinh\frac{t-z}{\lambda_S}}{\sigma_{\mathrm{Pt}}+2\lambda_S G_{\mathrm{r}}\coth\frac{t}{\lambda_S}}\right] \tag{S17}$$

Integral of the spin current over the thickness of the Pt layer, the longitudinal resistivity can be expanded with respect to the magnetization direction as

$$\rho_{\mathrm{long}}=\sigma^{-1}=\left(\frac{\bar{j_C}}{E_x}\right)^{1}\cong\rho+\Delta\rho_0+\Delta\rho_1\left(1-m_y^2\right) \tag{S18}$$

where

$$\frac{\Delta\rho_0}{\rho} = -\left(2\theta_{\mathrm{SH}} - \frac{\sigma_{\mathrm{CuO_x}}}{\sigma_{\mathrm{Pt}}}\theta_{\mathrm{OH}}\right)\theta_{\mathrm{SH}}\frac{\lambda_S}{t}\tanh\frac{t}{2\lambda_S} \tag{S19}$$

$$\frac{\Delta\rho_1}{\rho} = \left(\theta_{\mathrm{SH}}\tanh\frac{t}{2\lambda_S} + \frac{\sigma_{\mathrm{CuO_x}}}{\sigma_{\mathrm{Pt}}}\theta_{\mathrm{OH}}\,\mathrm{csch}\frac{t}{\lambda_S}\right)\theta_{\mathrm{SH}}\frac{\lambda_S}{t}\frac{2\lambda_S G_{\mathrm{r}}\tanh\frac{t}{2\lambda_S}}{\sigma_{\mathrm{Pt}} + 2\lambda_S G_{\mathrm{r}}\coth\frac{t}{\lambda_S}} \tag{S20}$$

In the main text, we use Eq. S20 to obtain the orbital Hall current generation from the $\mathrm{CuO_x}$ layer.

<u>Calculation of the magneto-thermopower (MTP)</u>

For the calculation of the MTP, we have $\nabla T \neq 0$ but $j_C = 0$. This result in the thermal electric field $E_{\mathrm{th}} = \partial_x\mu_0 = S\partial_x T - \theta_{\mathrm{SH}}\partial_z\mu_S^y/2e$ and the spin current $j_{Sz}^y = \sigma_{\mathrm{Pt}}(-\theta_{\mathrm{SN}}S\partial_x T - \partial_z\mu_S/2e)$. Similar to the case of MR, for MTP, the spin current at the YIG / Pt interface depends on the magnetization direction of YIG, while the spin current at Pt/$\mathrm{CuO_x}$ interface, $j_{Sz}^y(z=t) = \theta_{\mathrm{ON}}\sigma_{\mathrm{Cu\;\;x}}S_{\mathrm{Cu\;\;x}}\partial_x T$, does not depend on the magnetization. Using these boundary condition, solving A and B factors, we obtain the spin accumulation

$$\begin{aligned}\mu_S(z) = \frac{2e\lambda_S\partial_x T}{\sigma_{Pt}\sinh\frac{t}{\lambda_S}}\Bigg[&\sigma_{\mathrm{CuO_x}}S_{\mathrm{CuO_x}}\theta_{\mathrm{ON}}\cosh\frac{z}{\lambda_S} + \sigma_{\mathrm{Pt}}S_{\mathrm{Pt}}\theta_{\mathrm{SN}}\left(\cosh\frac{t-z}{\lambda_S} - \cosh\frac{z}{\lambda_S}\right)\\ &- m\times(m\times\hat{y})\left(\sigma_{\mathrm{CuO_x}}S_{\mathrm{CuO_x}}\theta_{\mathrm{ON}}\,\mathrm{csch}\frac{t}{\lambda_S} + \sigma_{\mathrm{Pt}}S_{\mathrm{Pt}}\theta_{\mathrm{SN}}\tanh\frac{t}{2\lambda_S}\right)\frac{2\lambda_S G_{\mathrm{r}}\cosh\frac{t-z}{\lambda_S}}{\sigma_{\mathrm{Pt}} + 2\lambda_S G_{\mathrm{r}}\coth\frac{t}{\lambda_S}}\Bigg]\end{aligned} \tag{S21}$$

spin current

$$j_{Sz}^{y}(z) = -\frac{2\partial_x T}{\sinh\frac{t}{\lambda_S}}\left[\sinh\frac{z}{2\lambda_S}\left(\sigma_{\mathrm{CuO_x}}S_{\mathrm{Cu\ \ x}}\theta_{\mathrm{ON}}\cosh\frac{z}{2\lambda_S} + \sigma_{\mathrm{Pt}}S_{\mathrm{Pt}}\theta_{\mathrm{SN}}\left(\cosh\frac{2t-z}{2\lambda_S} - \cosh\frac{z}{2\lambda_S}\right)\right)\right.$$
$$\left. + m\times(m\times\hat{y})\left(\sigma_{\mathrm{Cu\ \ x}}S_{\mathrm{Cu\ \ x}}\theta_{\mathrm{ON}}\,\mathrm{csch}\frac{t}{\lambda_S} + \sigma_{\mathrm{Pt}}S_{\mathrm{Pt}}\theta_{\mathrm{SN}}\tanh\frac{t}{2\lambda_S}\right)\frac{\lambda_S G_{\mathrm{r}}\sinh\frac{t-z}{\lambda_S}}{\sigma_{\mathrm{Pt}} + 2\lambda_S G_{\mathrm{r}}\coth\frac{t}{\lambda_S}}\right] \quad \text{(S22)}$$

and the thermal electric field

$$\sigma_{\mathrm{Pt}}E = \sigma_{\mathrm{Pt}}S_{\mathrm{Pt}}\partial_x T + \frac{\partial_x T}{\sinh\frac{t}{\lambda_S}}\left[-\sigma_{\mathrm{Cu\ \ x}}S_{\mathrm{Cu\ \ x}}\theta_{\mathrm{ON}}\theta_{\mathrm{SH}}\sinh\frac{z}{\lambda_S} + \sigma_{\mathrm{Pt}}S_{\mathrm{Pt}}\theta_{\mathrm{SN}}\theta_{\mathrm{SH}}\left(\sinh\frac{t-z}{\lambda_S} + \sinh\frac{z}{\lambda_S}\right)\right.$$
$$\left. -\left(1-m_y^2\right)\left(\sigma_{\mathrm{Cu\ \ x}}S_{\mathrm{CuO_x}}\theta_{\mathrm{ON}}\,\mathrm{csch}\frac{t}{\lambda_S} + \sigma_{\mathrm{Pt}}S_{\mathrm{Pt}}\theta_{\mathrm{SN}}\tanh\frac{t}{2\lambda_S}\right)\theta_{\mathrm{SH}}\frac{2\lambda_S G_{\mathrm{r}}\sinh\frac{t-z}{\lambda_S}}{\sigma_{\mathrm{Pt}} + 2\lambda_S G_{\mathrm{r}}\coth\frac{t}{\lambda_S}}\right] \quad \text{(S23)}$$

Integral of the thermally induced electric field over the thickness of the Pt layer, we could expand the Seebeck coefficient as

$$S_{xx} = S + \Delta S_0 + \left(1-m_y^2\right)\Delta S_1 \quad \text{(S24)}$$

Where

$$\frac{\Delta S_0}{S} = \left(2\theta_{\mathrm{SN}} - \frac{\sigma_{\mathrm{CuO_x}}S_{\mathrm{CuO_x}}}{\sigma_{\mathrm{Pt}}S_{\mathrm{Pt}}}\theta_{\mathrm{ON}}\right)\theta_{\mathrm{SH}}\frac{\lambda_S}{t}\tanh\frac{t}{2\lambda_S} \quad \text{(S25)}$$

$$\frac{\Delta S_1}{S} = -\left(\theta_{\mathrm{SN}}\tanh\frac{t}{2\lambda_S} + \frac{\sigma_{\mathrm{CuO_x}}S_{\mathrm{CuO_x}}}{\sigma_{\mathrm{Pt}}S_{\mathrm{Pt}}}\theta_{\mathrm{ON}}\,\mathrm{csch}\frac{t}{\lambda_S}\right)\theta_{\mathrm{SH}}\frac{\lambda_S}{t}\frac{2\lambda_S G_{\mathrm{r}}\tanh\frac{t}{2\lambda_S}}{\sigma_{\mathrm{Pt}} + 2\lambda_S G_{\mathrm{r}}\coth\frac{t}{\lambda_S}} \quad \text{(S26)}$$

We use Eq. S26 to calculate the orbital current induced from the orbital Nernst effect from CuO_x in the main text.

Supplementary Note 7. Contributions from the Pt/Cu interface.

Given that the CuO_x exhibits a non-uniform oxygen distribution, with higher oxygen content near the surface and lower content toward the bottom, the Pt/CuO_x interface can be approximated as a Pt/Cu-like interface.

To further examine the interfacial contribution, we performed a series of measurements using a 2-nm-thick Pt layer. As shown in Fig. S7(a), the YIG/Pt(2)/SiO_2(2) sample exhibits a magnetoresistance (MR) ratio of 2.43×10^{-4}. For the YIG/Pt(2)/Cu(2)/SiO_2(2) sample, shown in Fig. S7(b), the MR ratio decreases to 9.81×10^{-5}. In contrast, the YIG/Pt/CuO_x(2)/SiO_2(2) sample shows an enhanced MR ratio of 3.08×10^{-4}.

These results indicate that the Pt/Cu interface is unlikely to be responsible for the observed enhancement of the MR ratio. Therefore, we attribute the enhanced spin current in the YIG/Pt/CuO_x/SiO_2 sample primarily to the Cu-O bonding.

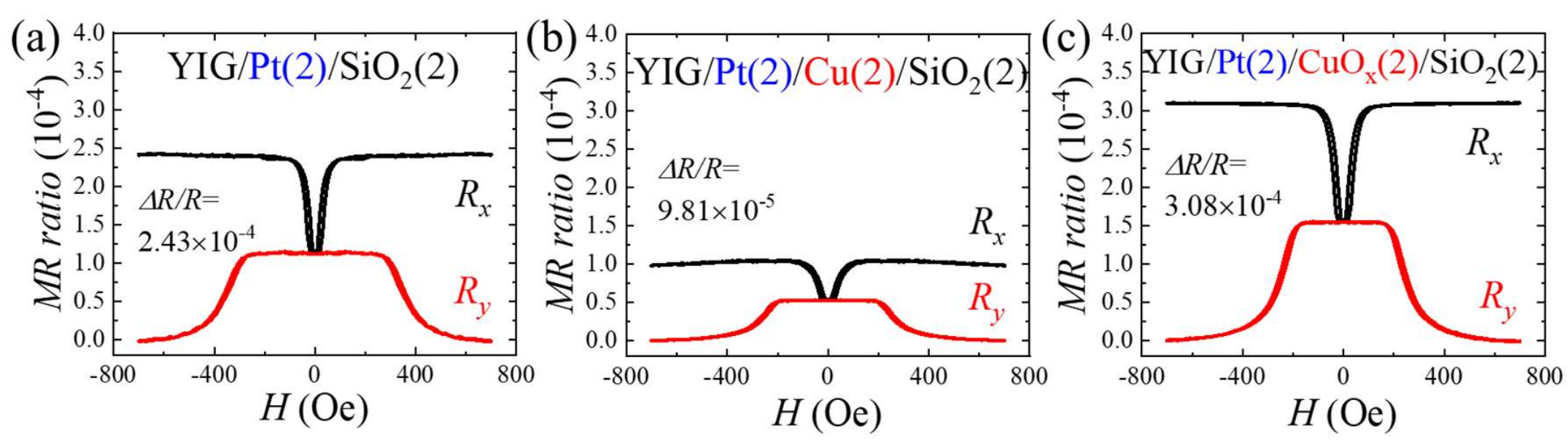

Fig. S7. Magnetoresistance (MR) ratios for (a) YIG/Pt(2)/SiO_2(2), (b) YIG/Pt(2)/Cu(2)/ SiO_2(2), and (c) YIG/Pt(2)/CuO_x(2)/SiO_2(2) samples.

References

[1] S. Meyer, Y.-T. Chen, S. Wimmer, M. Althammer, T. Wimmer, R. Schlitz, S. Geprägs, H. Huebl, D. Ködderitzsch, H. Ebert, G. E. W. Bauer, R. Gross and S. T. B. Goennenwein, *Observation of the spin Nernst effect*, Nat. Mater. **16**, 977 (2017).

[2] W.-Y. Li, C.-H. Lin, G.-Y. Guo, S.-Y. Huang, and D. Qu, *Large spin Nernst effect in the* $Ni_{70}Cu_{30}$ *alloy*, Phys. Rev. B **111**, 054421 (2025).

[3] W. Haynes, *CRC handbook of chemistry and physics*, CRC Press, (2014).

[4] Y.-T. Chen, S. Takahashi, H. Nakayama, M. Althammer, S. T. B. Goennenwein, E. Saitoh, and G. E. W. Bauer, *Theory of spin Hall magnetoresistance*, Phys. Rev. B **87**, 144411 (2013).
