# Supplementary material for: Transport evidence for the orbital Nernst effect
Source: Nat Commun. 2026 Sep 22;17:11032. doi: 10.1038/s41467-026-77878-2 (PMC13686958; doi:10.1038/s41467-026-77878-2)
Supplement: Supplementary file 2 [file 41467_2026_77878_MOESM2_ESM.pdf]

nature portfolio

Peer Review File

Transport Evidence for the Orbital Nernst Effect

Corresponding Author: Dr Danru Qu

This manuscript has been previously reviewed at another journal. This document only contains information relating to versions considered at Nature Communications.

This file contains all reviewer reports in order by version, followed by all author rebuttals in order by version.

Version 1:

Reviewer comments:

Reviewer #1

(Remarks to the Author)
I appreciate Wen-Yuan Li and co-authors revised their manuscript and provided a point-by-point response to the referees' comments. However, I have not been convinced by the authors' explanation yet.

As the authors added in the revised manuscript, the magnitude and the sign of Seebeck coefficient (S) are often "enhanced surface scattering when the film thickness approaches the electron mean free path" (on page 10). This fact also suggests that some "interfaces" also affect the magnitude and the sign of S. That means the formation of Pt / CuOx interface also changes the S. In addition, such interfaces may affect the magnitude of spin Hall effect for the spin Nernst effect. I don't think these comparative experiments between YIG/Pt(3)/CuOx(2)/SiO2(2) and YIG/Pt(3)/SiO2(2) alone can dispel my concern.
In the revised manuscript and supplementary information, the authors added the new MR measurement using the samples with Pt layer thickness of 2 nm. I feel that is partially useful information, but not enough. I think that it would be very helpful for revealing the underlying mechanism if the authors carry out more systematic Pt layer thickness dependence of thermoelectric measurement. It will give the more convincing experimental facts whether spin or orbital is dominant. If the orbital Nernst effect is dominant, the authors will observe the local maximum of the signal at a certain Pt layer thickness.

In conclusion, I do not change my opinion and cannot recommend the publication at the present form.

Reviewer #2

(Remarks to the Author)
The authors have addressed all my questions and comments. I recommend the publication of this paper in Nature Communications.

Reviewer #3

(Remarks to the Author)
The authors studied orbital Nernst magneto-thermopower in YIG/Pt/CuOx structures.Orbital Nernst contribution is suggested by the difference between different stacking structures. While providing first transport evidence for ONE,I agree with the previous reviewers that this cannot support the claim that the authors observed ONE, which should be claimed only after some direct evidence, e.g., MOKE measurements, is provided for an orbital current related to a thermal gradient.
Thermal transport in nanometer thin films itself is a large direction, containing intricate phenomena. For multilayer YIG/Pt/CuOx, it's also probably hard to clarify if authors' model can catch all the essential parts for the thermal transport.
Given that no experimental evidence for ONE has been revealed, I think this work is worth to be published in Nat. Commun. after removing these over claims and restricting their discovery in the transport field.

Version 2:

Reviewer comments:

Reviewer #1

(Remarks to the Author)
Wen-Yuan Li and co-authors did the additional experiments and revised their manuscript. I appreciate their great effort. Although they put the additional data for Pt layer thickness dependence in the Supplementary Information, I feel these are very important results to claim the ""transport evidence for the orbital Nernst effect", so I recommend showing the Pt layer thickness dependence in the main text as main figures. After their consideration of my suggestion, I am happy to recommend publication.

Reviewer #3

(Remarks to the Author)
The authors have addressed the overclaims in the previous manuscript as requested. Furthermore, following Reviewer 1's suggestion, they have examined how the MR and MTR ratios vary with thickness, which lends additional support to the orbital Nernst effect. I am pleased to recommend this manuscript for publication in its present form.

Open Access This Peer Review File is licensed under a Creative Commons Attribution 4.0 International License, which permits use, sharing, adaptation, distribution and reproduction in any medium or format, as long as you give appropriate credit to the original author(s) and the source, provide a link to the Creative Commons license, and indicate if changes were made.
In cases where reviewers are anonymous, credit should be given to 'Anonymous Referee' and the source.
The images or other third party material in this Peer Review File are included in the article's Creative Commons license, unless indicated otherwise in a credit line to the material. If material is not included in the article's Creative Commons license and your intended use is not permitted by statutory regulation or exceeds the permitted use, you will need to obtain permission directly from the copyright holder.
To view a copy of this license, visit https://creativecommons.org/licenses/by/4.0/

We would like to thank all reviewers for the constructive comments on our manuscript titled "*Observation of the Orbital Nernst Effect*". The updated manuscript and supplementary materials have benefited from reviewers' constructive suggestions. In this response letter, we provide point-to-point responses to the reviewers' comments and outline the changes made to the manuscript. The comments from the referees are in *black italic,* while our responses are in blue.

Referee #1:

I appreciate Wen-Yuan Li and co-authors revised their manuscript and provided a point-by-point response to the referees' comments. However, I have not been convinced by the authors' explanation yet.

As the authors added in the revised manuscript, the magnitude and the sign of Seebeck coefficient (S) are often "enhanced surface scattering when the film thickness approaches the electron mean free path" (on page 10). This fact also suggests that some "interfaces" also affect the magnitude and the sign of S. That means the formation of Pt / CuOx interface also changes the S. In addition, such interfaces may affect the magnitude of spin Hall effect for the spin Nernst effect. I don't think these comparative experiments between YIG/Pt(3)/CuOx(2)/SiO2(2) and YIG/Pt(3)/SiO2(2) alone can dispel my concern.

In the revised manuscript and supplementary information, the authors added the new MR measurement using the samples with Pt layer thickness of 2 nm. I feel that is partially useful information, but not enough. I think that it would be very helpful for revealing the underlying mechanism if the authors carry out more systematic Pt layer thickness dependence of thermoelectric measurement. It will give the more convincing experimental facts whether spin or orbital is dominant. If the orbital Nernst effect is dominant, the authors will observe the local maximum of the signal at a certain Pt layer thickness.

Response:

We thank the referee for this valuable suggestion. We agree that Pt-thickness-dependent measurements are important for examining the respective contributions associated with the Pt layer, the CuO_x layer, and the interfaces. Following the referee's suggestion, we have

performed a comparative analysis of the Pt-thickness-dependent results for the YIG/Pt(t)/SiO_2(2) and YIG/Pt(t)/CuO_x(2)/SiO_2(2) samples.

First, we find that for the YIG/Pt(t)/SiO_2 reference samples, the Seebeck coefficient of Pt varies substantially with Pt thickness as shown in Fig. R1(a), confirming the strong thickness dependence of the Seebeck coefficient in ultrathin films. However, the magneto-thermopower ratio exhibits a much weaker thickness dependence and does not display a corresponding enhancement in the thin-Pt regime, as shown in Fig. R1(b). Thus, the change of the Seebeck coefficient alone does not suggest a higher spin Nernst contribution.

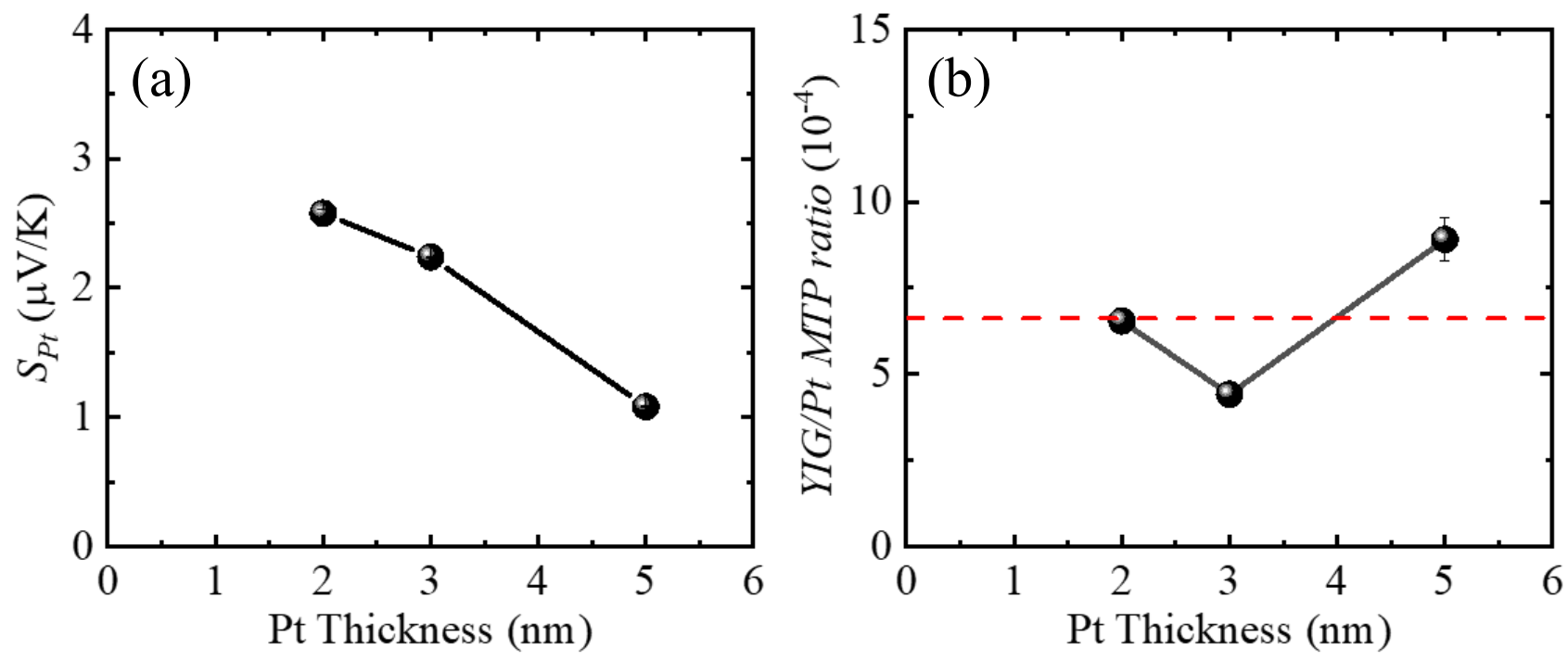

Fig. R1. (a) Pt-thickness dependent Seebeck coefficient. (b) Pt-thickness dependent magneto-thermopower ratio of the YIG/Pt(t)/SiO_2(2) sample.

To further address the referee's concern, we compare the effective thickness-dependent magnetoresistance (MR) and magneto-thermopower (MTP) ratios of the Pt layer for the YIG/Pt(t)/SiO_2(2) and YIG/Pt(t)/CuO_x(2)/SiO_2(2) samples, as shown by the black and red symbols, in Fig. R2(a) and R2(b). For the samples containing CuO_x(2), both the MR and MTP ratios increase markedly as the Pt thickness is reduced from 5 nm to 2 nm. By comparison, the corresponding thickness dependence in the YIG/Pt(t)/SiO_2 reference samples is considerably weaker, particularly for the MTP ratio, as shown in Fig. R2(b).

The contrasting thickness dependence observed in the samples with and without CuO_x are consistent with the behavior reported in Phys. Rev. Lett. **125**, 177201 (2020), where the signals of the CuO_x-capped and uncapped samples diverged in the thin-Pt regime and gradually converged as the Pt thickness increased. In that study, this additional contribution was attributed to orbital-angular-momentum generation associated with the CuO_x, followed by

orbital-to-spin conversion in Pt. Accordingly, the different Pt-thickness-dependent MTP ratio for the YIG/Pt(t)/SiO_2(2) and YIG/Pt(t)/CuO_x(2)/SiO_2(2) samples also support the presence of an additional orbital-current contribution generated by the in-plane thermal gradient in CuO_x.

Taken together, the Pt-thickness-dependent Seebeck results show that the enhanced MTP ratio in the CuO_x-containing samples cannot be accounted for by the change of the Seebeck coefficient alone. The parallel enhancement of the MR and MTP ratios in the thin-Pt regime suggests an additional contribution associated with the CuO_x layer. Furthermore, the different thickness dependences for both the MR and MTP results support the contribution from orbital current generation in CuO_x followed by orbital to spin conversion in Pt. These results provide additional evidence for orbital Nernst effect in CuO_x.

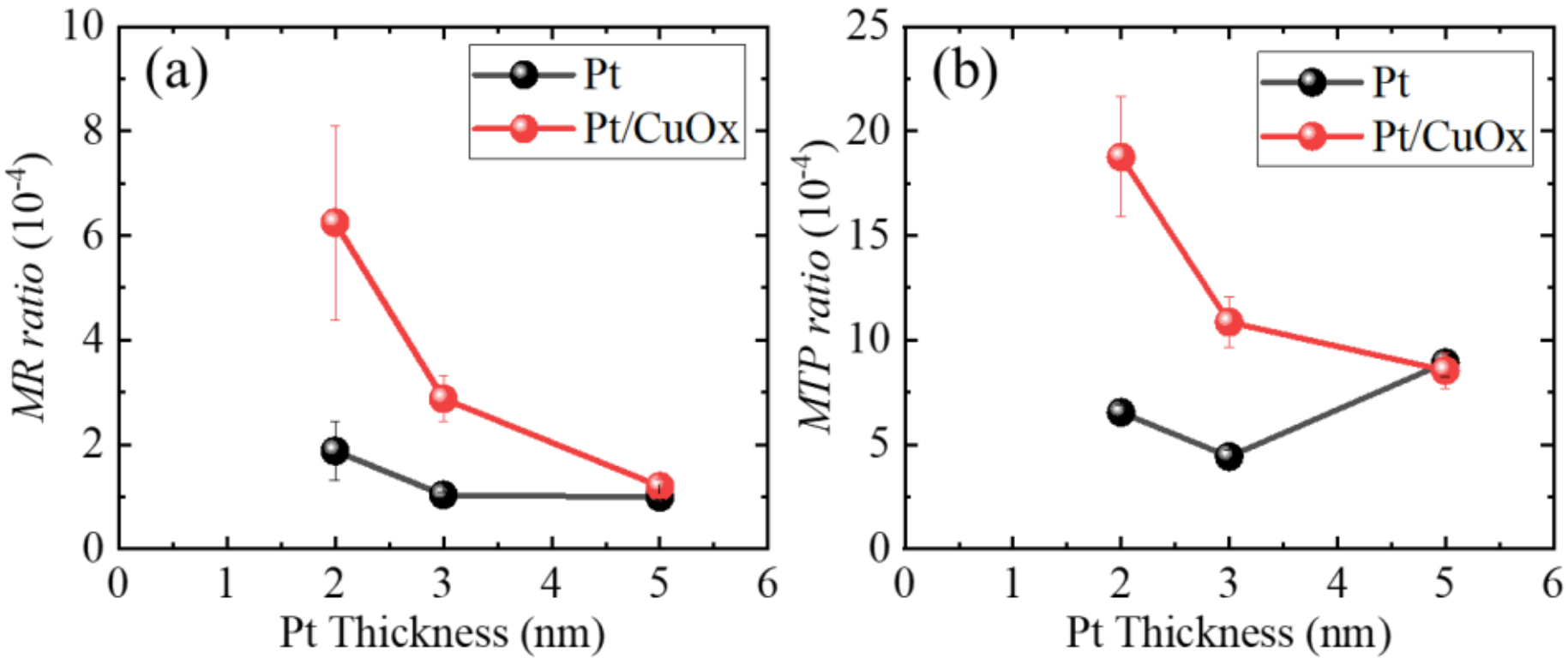

Fig. R2. Thickness-dependent effective (a) MR ratio and (b) MTP ratio for Pt in YIG/Pt(t)/SiO_2(2) (black) and YIG/Pt(t)/CuO_x(2)/SiO_2(2) (red) samples.

Thanks to Referee 1's suggestion, we have now included these Pt-thickness dependent results in the supplementary materials as Supplementary Information S8, and we added a paragraph describing the results in the main text on page 17 as

"*A more detailed comparison for YIG/Pt(t)/SiO*$_2$*(2) and YIG/Pt(t)/CuO*$_x$*(2)/SiO*$_2$*(2) samples with t ranging from 2 to 5 nm is shown in Supplementary Information S8.*"

In addition, combining with Referee 3's suggestion, we have further revised the manuscript throughout to avoid overstating our conclusions. Specifically, statements such as "observation of the orbital Nernst effect" have been replaced with more careful descriptions,

including "transport evidence for the orbital Nernst effect", "an orbital Nernst contribution", and "results consistent with a thermally generated orbital current". To further reflect the novelty and also limitation of our findings, we have revised the title to: "Transport Evidence for the Orbital Nernst Effect."

In conclusion, I do not change my opinion and cannot recommend the publication at the present form.

Response:

We thank the referee for the constructive and rigorous review.

Referee #2:

The authors have addressed all my questions and comments. I recommend the publication of this paper in Nature Communications.

Response:

We thank the referee for the careful evaluation of our revised manuscript and for the positive recommendation. We greatly appreciate the referee's constructive comments during the review process, which have helped us improve the clarity and quality of the manuscript.

Referee #3:

The authors studied orbital Nernst magneto-thermopower in YIG/Pt/CuOx structures. Orbital Nernst contribution is suggested by the difference between different stacking structures. While providing first transport evidence for ONE, I agree with the previous reviewers that this cannot support the claim that the authors observed ONE, which should be claimed only after some

direct evidence, e.g., MOKE measurements, is provided for an orbital current related to a thermal gradient.

Thermal transport in nanometer thin films itself is a large direction, containing intricate phenomena. For multilayer YIG/Pt/CuOx, it's also probably hard to clarify if authors' model can catch all the essential parts for the thermal transport.

Given that no experimental evidence for ONE has been revealed, I think this work is worth to be published in Nat. Commun. after removing these over claims and restricting their discovery in the transport field.

Response:

We thank the referee for the careful assessment of our revised manuscript and for recognizing the significance of the transport evidence presented in this work. We also appreciate the referee's constructive recommendation that the scope of our claims should be limited to transport evidence for the orbital Nernst effect rather than direct observation.

Following the referee's suggestion, we have revised the manuscript throughout to avoid overstating our conclusions. Specifically, statements such as "observation of the orbital Nernst effect" have been replaced with more careful descriptions, including "transport evidence for the orbital Nernst effect", "an orbital Nernst contribution", and "results consistent with a thermally generated orbital current". To further reflect the scope of our findings, we have revised the title to:

"Transport Evidence for the Orbital Nernst Effect"

In addition, we have revised the Abstract, Introduction, Discussion, and Conclusion to consistently emphasize that the primary contribution of this work is the identification of an additional magneto-thermopower signal in YIG/Pt/CuO_x/SiO_2(2) heterostructures that is consistent with the picture where an orbital current is generated in CuO_x under a heat current and subsequently converted into a spin current in Pt through orbital-to-spin conversion. We believe these revisions reflect both the strengths and the limitations of the present work while preserving its novelty as the first transport evidence for the orbital Nernst effect.

We would like to thank all reviewers for the constructive comments on our manuscript titled "*Transport Evidence for the Orbital Nernst Effect*". The updated manuscript and supplementary materials have benefited from reviewers' constructive suggestions. In this response letter, we provide point-to-point responses to the reviewers' comments and outline the changes made to the manuscript. The comments from the referees are in *black italic,* while our responses are in blue.

Referee #1:

Wen-Yuan Li and co-authors did the additional experiments and revised their manuscript. I appreciate their great effort. Although they put the additional data for Pt layer thickness dependence in the Supplementary Information, I feel these are very important results to claim the ""transport evidence for the orbital Nernst effect", so I recommend showing the Pt layer thickness dependence in the main text as main figures. After their consideration of my suggestion, I am happy to recommend publication.

Response:

We thank the reviewer for the positive assessment of our revised manuscript and for this constructive suggestion. We agree that the Pt-thickness-dependent measurements provide important support for the orbital Nernst effect and should be presented more prominently. We have therefore moved this dataset from the Supplementary Information (previously Section S8) to the main text as a new Fig. 5, which shows the Pt-thickness dependence of the Seebeck coefficient and magneto-thermopower ratio [Figs. 5(a)-(b)], as well as the effective magnetoresistance and magneto-thermopower ratios for the YIG/Pt(*t*) and YIG/Pt(*t*)/CuO_x(2) samples [Figs. 5(c)-(d)]. We have revised the corresponding text in the Results section to present and discuss these results in the main text, and we have also updated the Summary to explicitly reference these findings. The relevant Supplementary Information section has been removed accordingly. We believe this revision better highlights the importance of these results in supporting our conclusions, as suggested by the reviewer.

Referee #3:

The authors have addressed the overclaims in the previous manuscript as requested. Furthermore, following Reviewer 1's suggestion, they have examined how the MR and MTR ratios vary with thickness, which lends additional support to the orbital Nernst effect. I am pleased to recommend this manuscript for publication in its present form.

Response:

We thank the reviewer for the positive assessment and recommendation to publish the manuscript in its present form.
